# Nephroprotective effect of *Physalis peruviana* L. calyx extract and its butanolic fraction against cadmium chloride toxicity in rats and molecular docking of isolated compounds

**DOI:** 10.1186/s12906-023-03845-9

**Published:** 2023-01-27

**Authors:** Hesham S. M. Soliman, Eslam M. Korany, Elsayed K. El-Sayed, Ahmed M. Aboelyazed, Haitham A. Ibrahim

**Affiliations:** 1 Department of Pharmacognosy, Faculty of Pharmacy, Helwan University, Ain Helwan, Cairo, 11795 Egypt; 2 Pharm D program, Egypt-Japan University of Science and Technology, New Borg El-Arab City, 21934 Egypt; 3 Department of Pharmacology and Toxicology, Faculty of Pharmacy, Helwan University, Ain Helwan, Cairo, 11795 Egypt; 4 Department of Pharmaceutical Chemistry, Faculty of Pharmacy, Badr University, Badr City, 11829 Cairo Egypt

**Keywords:** Anti-inflammatory, Antioxidant, Nephrotoxicity, *Physalis peruviana* L calyx, Withanolides

## Abstract

**Background:**

Cadmium is an environmentally toxic metal that has deleterious effects on both animals and humans due to its accumulation in different body tissues. *Physalis peruviana* L. fruit and calyx contain many active constituents which are used traditionally for their different biological activities. Based on the traditional uses of *P. peruviana* L. calyx, we aimed to evaluate the nephroprotective effect of their 80% aqueous methanol extract (AME) and n-butanol fraction (Bu.F.) against cadmium chloride-induced nephrotoxicity in rats and to correlate this activity with phytoconstituents isolated using molecular docking studies.

**Methods:**

The n-butanol fraction of *P. peruviana* L. calyx was fractionated using various chromatographic techniques and the isolated compounds were identified based on their chemical and spectroscopic data. The nephroprotective activity was assessed using cadmium chloride-induced nephrotoxicity in the rat model, by measuring some important parameters such as body weight, kidney weight, serum urea, and creatinine levels, oxidative stress markers, inflammatory markers, and histopathological examinations of kidney tissue. Molecular docking studies of the isolated compounds were performed.

**Results:**

Three withanolides named 4 β-hydroxywithanolide E (1), Physalin B (2) and 3*α*, 14*β*-dihydroxywithaphysalin N (3) were isolated and identified from the *n*-butanol fraction of *P. peruviana* L calyx extract. The extract and its butanol fraction significantly improved the serum kidney function markers and tissue oxidative status including malondialdehyde (MDA), reduced glutathione (GSH) and catalase (CAT). Additionally, the extracts significantly decreased the levels of tumor necrosis factor-alpha (TNF-α) and nuclear factor kappaB (NF-κβ). Moreover, the histological changes were ameliorated by the extracts. The molecular docking study showed that the isolated compounds displayed a remarkable inhibitory activity against IκB kinase.

**Conclusion:**

The AME and its butanol fraction of *P. peruviana* L calyx showed potential nephroprotective activity against cadmium chloride-induced nephrotoxicity which is correlated at least in part to its considerable withanolides content.

**Supplementary Information:**

The online version contains supplementary material available at 10.1186/s12906-023-03845-9.

## Background

*Physalis* is a genus of herbaceous plants belonging to the family Solanaceae. Such genus contains approximately 120 species, mainly distributed in tropical and temperate regions. Most species of *Physalis* have been used for a long time in folk medicine to treat different illnesses, such as dermatitis, hepatitis, liver disorders, malaria, asthma, *mycobacterium*, leukemia, and as antipyretic, anticancer, and immuno-modulatory agent [1].

*Physalis peruviana* L. fruit grows wild in South America at high altitudes in tropical Colombia, Ecuador, Chile, and Peru. Nowadays, it is cultivated in many different climatic regions. The fruit is yellow and surrounded by papery calyx resembling tomato in appearance with a sweet and sour taste. The fruits are eaten raw or used in salads, cocktails, jams, jellies, ice cream and desserts [2]. The fruit contains a lot of valuable constituents like minerals, carbohydrates, lipids, flavonoids, polyphenols, vitamins, organic acids, withanolides, carotenoids, flavoring compounds and phytosterols [3]. The fruit has diverse activities as antioxidant, anti-microbial activities [4], anti-cancer [5], renoprotective [6], hypoglycaemic [7], anti-inflammatory [8] and anti-hypocholesteremia [9]. Despite its therapeutic benefits, it is still cultivated in a few parts of Egypt as an edible plant and no attention has been paid to utilize it in local food industries [10].

The calyx is the largest by-product produced during harvesting and selling the fruit. They contain many chemical constituents that possess different biological activities as anti-inflammatory, antioxidant [11], antihyperlipidemic [12], hypoglycemic [13] and anticancer activity [14].

The kidney is one of the most important excretory organs; besides maintaining physiological fluids, electrolytes balance and controlling blood pressure through hormonal secretions. Toxic compounds in the blood circulation may react with the arteries, glomeruli, and tubules of the kidneys, resulting in changes in anatomy and renal functions [15]. Kidney injury or failure can be classified as acute or chronic depending on the causes and the duration of injury; can lead to partial or complete loss of kidney functions where the only solution is dialysis or kidney transplantation [16]. Cadmium (Cd) is one of the most essential reactive toxic metals that have been significantly increased in the environment from both anthropogenic and natural sources. Contaminated soil, water, air, cigarette and food are also major sources of Cd exposure [17]. Exposure to Cd is associated with multiorgan damage including liver, kidney, brain, lung, heart, and bone in both animals and humans. Among these organs, the kidney is the most susceptible organ to chronic Cd exposure due to its vast accumulation in the renal glomeruli and proximal tubules resulting in nephrotoxicity [18]. At the molecular level, accumulation of Cd in renal tissues induces oxidative stress and generation of reactive oxygen species (ROS) which is accompanied by inflammation and apoptosis [19, 20].

The present study aims to isolate some promising withanolides and evaluate the nephroprotective effect of *P. peruviana* L. calyx 80% aqueous methanolic extract and the *n*-butanol fraction. Molecular docking was applied to evaluate and assure the binding affinity and intermolecular interactions of the isolated compounds to predict and correlate the activity of the extracts to the isolated compounds.

## Methods

### Instruments and material

The 1D and 2D-NMR spectra were recorded on Bruker Avance DRX (Bruker, Rheinstetten, Germany) at 400 MHz (for ^1^H NMR) and 100.40 MHz (for ^13^C NMR, DEPT and 2D). Each sample should be dried using a rotary evaporator before being submitted to the NMR measurement and is then dissolved in a suitable NMR deuterated solvent. The resulting NMR signals were reported in part per million (ppm) relative to tetramethylsilane (TMS) as an internal standard with coupling constants (*J* values) measured in Hz. ESI–MS was measured using GCT Premier Spectrometer (Waters, Milford, MA, USA). Silica gel (200–400 mesh, Merck, Darmstadt, Germany) and Sephadex LH-20 [25–100 µm] (Sigma-Aldrich-Steinhem, Germany) were used for column chromatography (CC), while DC Alufolien Silica gel 60 F_254_ (0.5 mm, Merck, Darmstadt, Germany) were used for preparative thin layer chromatography (PTLC). Detection of the column fractions, as well as pure samples, was done on TLC plates (Merck, Darmstadt, Germany) using *P*-anisaldehyde-sulfuric acid spray reagent [21] and visualization under UV light by UV lamp (Carl Roth GmbH and Co. KG, Germany). *n*-hexane, ethyl acetate, *n*-butanol, dichloromethane and methanol, were of analytical grade and purchased from El Nasr Pharmaceutical Chemicals Co, Egypt. Anhydrous cadmium chloride (CdCl_2_) of analytical grade was obtained from Sigma-Aldrich Chemical Co. (St. Louis, MO, USA). Silymarin tablets (South Egypt Drug Industries”SEDICO”, 6^th^ of October City, Egypt).

### Plant material

*Physalis peruviana* L. fresh fruits were collected from Nesmaya Farm, Shebin El-Qanater, Qalyubia, Egypt in April 2019. The plant was collected in accordance with national guidelines and regulations. It was kindly identified by Dr. Therese Labib, former plant taxonomy specialist at El Orman Botanical Garden, Giza, Egypt. A voucher specimen was kept in the herbarium of the Pharmacognosy department at Helwan University with the serial number 27 Ppe1 / 2019.

### Extraction and isolation

The calyx of *P. peruviana* L. was separated from 15 kg of fresh fruits, air-dried, and coarsely ground using a grinder. The powdered calyx (350 g) was firstly defatted with *n*-hexane thrice (3 × 3 L) and filtered. The defatted powdered calyx was allowed to dry and then macerated in 80% aqueous methanol (4 × 3 L) at room temperature. The methanol extract was dried under reduced pressure to obtain a total of 50 g crude aqueous methanol extract (AME) which has been reconstituted in distilled water (200 ml) and successively partitioned with ethyl acetate followed by *n*-butanol saturated with water each (3 × 200 ml). The ethyl acetate and *n*-butanol fractions (Bu. F.) were dried under reduced pressure to give 10 and 25 g residues respectively. Based on the TLC comparison, it was found that the *n*-butanol fraction is more promising. The *n*-butanol fraction: (25 g) was fractionated on a silica gel column (CC: Ø 4 × 70 cm; 500 g), eluted with dichloromethane: methanol mixture with increasing polarity (from 0%—50% methanol). The collected fractions were combined based on their similarity to TLC giving 6 main fractions (FI – FVI). The most interesting were FII and FIII. FII (700 mg) was then re-chromatographed on a silica column and eluted by (dichloromethane: methanol) (100:0 to 95:5). Seven sub-fractions were obtained (FIIa—FIIg). FIIc (55 mg), which had two major spots of two inseparable compounds, was purified on a Sephadex LH-20 column using 80% methanol, to give compounds 1 and 2 as a mixture (35 mg); whereas 1 was more abundant than 2. FIII (650 mg) was applied on a silica column using dichloromethane: methanol (100:0 to 90:10). Five sub-fractions were obtained (FIIIa—FIIIe). FIIId (80 mg) showed four spots. The most distinct one had been isolated by PTLC using (dichloromethane: methanol) (85:15 v/v) for development. This major band (25 mg) was further purified using Sephadex LH-20 column and methanol to afford the pure compound 3 (18 mg).

### Evaluation of the nephroprotective effect

#### Experimental animals

Adult male *Sprague Dawley* rats (180–220 g) and Swiss albino mice (20-25 g) were purchased from the breeding unit of the Egyptian Organization of Biological products and Vaccines (Helwan, Egypt). Animals were randomized and housed (3 rats/cage) in stainless steel wire bottom cages under controlled environmental conditions at a constant temperature (22ºC ± 2ºC). Animals were allowed free access to a standard pellet diet (Meladco Feed Company, Obour City, Cairo, Egypt) for two weeks acclimatization period before the experimental procedures and given tap water ad libitum. Animal care and experimental protocols were approved by the animal care and use committee at the Faculty of Pharmacy, Helwan University (Protocol No: 010A2021), and conducted according to the European Community Directive (68/609/EEC), a national rule on animal care that is consistent with the NIH Guidelines for the Care and Use of Laboratory Animals (8^th^ edition).

#### Determination of acute oral toxicity study (LD50)

Mice were administered orally with graded doses of AME and Bu.F. of P. peruviana L. up to 5 g/ kg. General animals’ behavior (activity, locomotion, pupil size, feeding and hair texture) as well as the percentage of mortality were observed over 24 h [22].

#### Experimental design

Forty-two rats were randomly divided into seven experimental groups (*n* = 6) and classified as follows:

- Group I: served as a control group.
- Group II: rats were administered distilled water orally for 10 days.
- Group III: rats received oral AME 500 mg/kg for 10 days.
- Group IV: rats received oral AME 1000 mg/kg for 10 days.
- Group V: rats administered Bu. F. 500 mg/kg orally for 10 days.
- Group VI: rats administered Bu. F. 1000 mg/kg orally for 10 days.
- Group VII: standard group, rats received oral silymarin 150 mg/kg for 10 days.

**Fig. 1:**
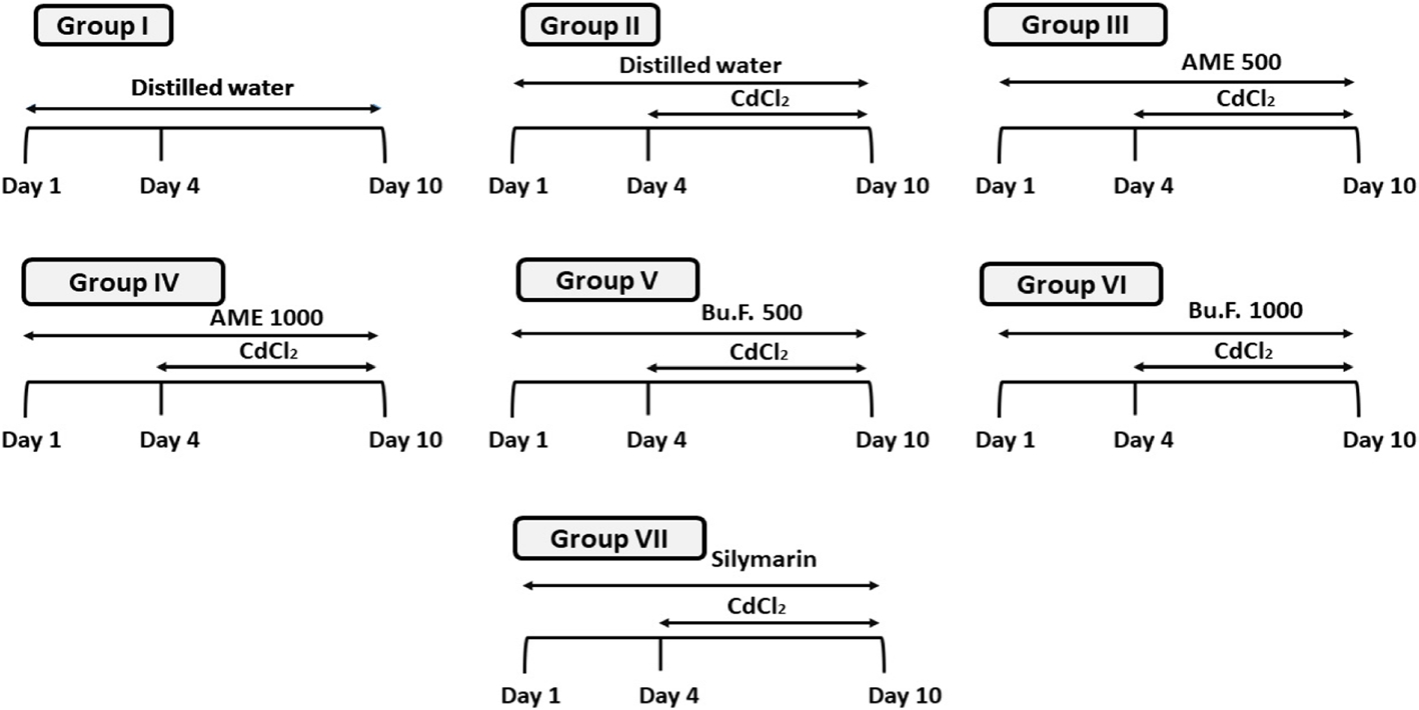
Schematic diagram for the experimental design. CdCl_2_: Cadmium chloride, AME: Aqueous methanolic extract, Bu.F: Butanol Fraction

From day 4 to day 10, 3 mg/kg CdCl2 were injected intraperitoneally to induce nephrotoxicity for all groups except the control group (Group I) (Fig. 1). Selections of CdCl_2_ and silymarin doses were based on previous studies [23, 24].

#### Blood samples and tissue collection

At the end of the experiment, rats were anaesthetized using sodium thiopental (40 mg/kg/ i.p.) [25]. Blood samples were collected from the retro-orbital plexus using non-heparinized tubes and allowed to coagulate at room temperature for 30 min and centrifuged at 3000 rpm for 30 min at 4 °C. The serum was stored at -20^o^ C for measuring kidney markers. Then, rats were killed by cervical dislocation. The abdomen of the rats were opened to quickly isolate all kidneys. For histopathological examinations, the right kidneys were post-fixed in 10% formal saline solution, while the left kidneys were homogenized in ice-cold phosphate buffer (0.1 M, pH 7.4) using an ice-cold Teflon homogenizer (Glas-Col®, USA) to obtain (10% w/v) homogenate that was centrifuged for 30 min at 3000 rpm and 4 oC. The homogenate obtained was used for the estimation of biochemical parameters.

### Biochemical assays

#### Serum markers of kidney dysfunction

The levels of urea and creatinine in serum were estimated spectrophotometrically using commercial diagnostic kits (Biodiagnostic, Egypt, Cat. No. UR 2110/CR 1251) respectively.

#### Measurement of oxidative stress markers in kidney tissues

The content of lipid peroxidation in kidney homogenates was quantified by measuring thiobarbituric acid reactive substances (TBARS) expressed as the amount of malondialdehyde (MDA) production using BioVision ELISA assay kit (BioVision, Milpitas, Cat. No. 95035, USA). The reduced glutathione (GSH) level in kidney homogenates was measured using Rat reduced glutathione ELISA kit (MyBioSource, San Diego, Cat. No. MBS724319, USA). The catalase (CAT) activity was measured according to Aebi et al., 1984 [26].

#### Measurement of inflammatory markers in the kidney tissues

The concentrations of tumor necrosis factor-alpha (TNF-α) and nuclear factor-kappa B (NF-κB) were determined in kidney homogenates using ELISA kits (MyBioSource, San Diego, Cat. No. MBS824824, USA), and (CUSABIO, Texas, Cat. No. CSB-E13148r, USA) respectively.

#### Histopathological examinations

The fixed kidney tissues were processed routinely, embedded in paraffin, sectioned, deparaffinized, rehydrated and stained with hematoxylin and eosin (H&E), using standard techniques [27].

### Molecular docking

Molecular docking is generally based on crystal structures of receptors or proteins with bound ligand molecules. The structure of the selected enzyme was determined using RCSB Protein Data Bank X-ray crystal data (PDB). Human IκB kinase was the selected target for the study. Co-crystallized ligand molecules are known drugs with proven action in the majority of selected proteins, which help in the determination of the binding site location as well as serving as references in our considerations.

CDOCKER protocol was performed for this study using Accelrys Discovery Studio® 2.5 software to achieve an overview of the SAR of the isolated compounds and to understand the results obtained by the biological evaluation based on ligand–protein interactions docking. This was achieved by extracting the ligand native to the active binding pockets of the IκB kinase enzyme and re-docking their various conformations again to the same binding site with less than 4 A°. The docking scores or Gibbs free energy (ΔG, kcal/mol) based on hydrogen bonds, hydrophobic and Vander Waal forces were the main criteria to rank the docking poses. The structures of the compounds were retained flexible to assume all possible conformations in the docking calculations. Detailed residue interaction analysis showed a difference in the affinities of these molecules according to the top-scoring pose of each molecule selected according to the best interaction energy.

### Statistical analysis

The results were expressed as mean value ± SEM. Statistical analysis and graphical representations were performed using GraphPad + Prism, version 8 (GraphPad Software Inc., San Diego, California, USA), using a one-way analysis of variance, followed by a Tukey’s test to determine the statistical significance between various groups. The value *p* < 0.05 was considered significant.

## Results

### Identification of the isolated compounds

**Fig. 2:**
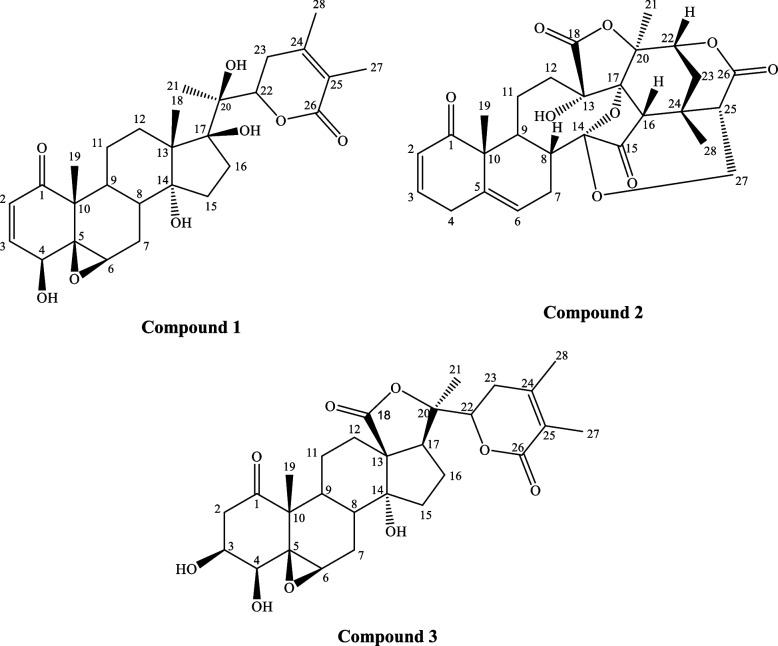
Withanolides isolated from *P. peruviana* L. calyx Bu. F

The applied chromatographic separation and purification methods resulted in three compounds. The separated compounds were structurally elucidated by different spectroscopic techniques (1D, 2D-NMR and ESI–MS). Compounds 1, 2, and 3 (Fig. 2) were obtained as pale-yellow solids. Compounds 1 and 2 displayed a dark color spot under short UV light on the TLC plate, which turned dark red after spraying with *p*-anisaldehyde**/**sulphuric acid spray reagent and heating at 120 °C, while compound 3 didn’t display any color under short or long UV light but gave dark pink spot after spraying with *p*-anisaldehyde**/**sulphuric spray reagent and heating at 120^o^ C.

#### Compound 1

^1^H NMR data of Compound 1 (Table 1) revealed that it possesses a withanolide skeleton; it displayed the presence of five methyl groups at δ ppm 1.18, 1.29, 1.26, 1.74, and 1.86 characteristics for H-18, 19, 21, 28 and 27 respectively. Moreover, the presence of a 5*β*, 6*β*-epoxy-4*β*-hydroxy-2-en-1-one unit in rings A and B was deduced from the signals at δ 6.08 (1H, d, *J* = 9.87), 6.96 (1H, dd, *J* = 9.2, 6.2), 3.56 (1H, d, *J* = 6.10) and 3.11 (brs) assigned to H-2, H-3, H-4 and H-6, respectively. H–H COSY spectrum (Table 1) of compound 1 showed a correlation between (H-2 and 3) and (H-3 and 4). The deduced pattern of rings A and B was finally confirmed by the carbon signals at δ ppm 202.82, 131.97, 143.66, 69.71, 63.41 and 60.57 attributed to C-1 to C-6, respectively. The ^13^C NMR data (Table 1) showed 28 carbon signals, including one carbonyl carbon (δ C 167.68) and two olefinic carbons (δ C 152.10 and 120.51); characteristic for the lactone ring of withanolides. DEPT data (Table 1) displayed six oxygenated carbons at (δ C 69.71, 63.41, 60.57, 82.46, 87.22 and 78.42) characteristic for C-4, C-5, C-6, C14, C-17 and C-20 respectively, and five methyl carbons at (δ C 19.21, 15.23, 18.03, 13.02, and 10.98) characteristic for C-18, C-19, C-21, C-27 and C-28 respectively. Also, a non-conjugated carbonyl carbon at δ C 202.82 (C-1) and a lactone carbonyl carbon at δ C 167.68 (C-26). All these features suggested a typical withanolide skeleton. ^1^H-^13^C HMBC long-range correlations (Table 1) confirmed the deduced structure. The positive ESI–MS spectrum of compound 1 gave a final confirmation of the structure that showed a molecular ion peak at *m/z* 503.176 [M + H]^+^, corresponding to the molecular weight 502.1. According to the above data and by comparison with previously reported data of related compounds [28–30]. Compound 1 is identified as 4 *β-*hydroxywithanolide E.

**Table 1:** ^1^H NMR (400 MHz), ^13^C NMR and DEPT (100 MHz), H–H COSY and HMBC spectroscopic data for Compound 1 in CD_3_OD

position	*δ*_H_ (mult, *J* in HZ)	*δ*_C_ (DEPT)	H–H COSY	HMBC
1		202.82		
2	6.08 (1H, d, J = 9.87)	131.97 (CH)	H-3, H-4	C-1, C-3, C-4, C-10
3	6.96 (1H, dd, J = 9.2, 6.2)	143.66 (CH)	H-2, H-4	C-1, C-4, C-5
4	3.56 (1H, d, J = 6.10)	69.71 (CH)	H-2, H3	C-2, C-3, C-10
5		63.41		
6	3.11 (1H, brs)	60.57 (CH)		C-4, C-7, C-8
7		29.09 (CH_2_)		
8		33.8 (CH)		
9		37.06 (CH)		
10		49.0		
11		25.81 (CH_2_)		
12		29.62 (CH_2_)		
13		54.11		
14		82.46		
15		31.80 (CH_2_)		
16		36.78 (CH_2_)		
17		87.22		
18	1.18 (3H, S)	19.21 (CH_3_)		
19	1.26 (3H, S)	15.23 (CH_3_)		
20		78.42		
21	1.29 (3H, S)	18.03 (CH_3_)		
22	4.80 (1H, m)	81.43 (CH)		
23		34.25 (CH_2_)		
24		152.10		
25		120.51		
26		167.68		
27	1.86 (3H, S)	13.02 (CH_3_)		
28	1.74 (3H, S)	10.98 (CH_3_)		

^1^H NMR (400 MHz); ^13^CNMR (100 MHz); *J*-value is expresses in Hz between parentheses

#### Compound 2

^1^H NMR data of Compound 2 indicated the presence of three singlet signals of tertiary methyl groups at δ ppm 1.30, 1.77 and 1.15 characteristic for H-19, 21 and 28 respectively, a pair of mutually coupled olefinic protons of a conjugated enone system at δ ppm 5.71 (dd, *J* = 10.02, 2.81 Hz) and 6.80 (ddd, *J* = 9.83, 4.70, 2.33 Hz) characteristic for H-2 and H-3 respectively. A further olefinic proton of a trisubstituted double bond at δ ppm (5.55, br d, J = 6 Hz) for H-6. A pair of characteristic signals at δ ppm (3.56, m and 4.68, m) assignable to the methylene protons at C-27 revealed that compound 2 possesses the C-27-O-C-14 bridge as present in physalins [31]. H–H COSY spectrum (Table 2) of compound 2 showed a correlation between (H-2 and 3) and (H-3 and 4) that confirmed the presence of a 2, 5-di en-1-one unit in rings A and B. The ^13^C NMR data (Table 2) showed 28 carbon signals, including two non-conjugated carbonyl carbon groups at δ ppm (205.39 and 211.15) for C-1 and C-15 respectively, two lactone groups at δ ppm (172.71 and 167.84) for C-18 and C-26 respectively and four olefinic carbons at δ ppm (126. 98, 146. 73, 135.34 and 124.79) characteristic for C-2, C-3, C-5 and C-6 respectively. Moreover, the ^13^C NMR and DEPT data (Table 2) indicated the presence of six methylene carbons at δ ppm (33.04, 25.29, 24.38, 28.82, 31.63 and 60.84) for C-4, C-7, C-11, C-12, C-23 and C-27 respectively, one ketal carbon at δ ppm 103.52 for C-14 and three methyl groups at δ ppm (19.61, 19.37 and 12.89) for C-19, C-21, and C-28 respectively. Consequently, the structure of compound 2 was deduced to be physalin B. ^1^H-^13^C HMBC long-range correlations (Table 2) confirmed the deduced structure. The positive ESI–MS spectrum of compound 2 showed a molecular ion peak at *m/z* 511.48 [M + H]^+^, corresponding to the molecular weight 510.54. According to the above-discussed data and by comparison with previously reported data of related compounds [32–35]. Compound 2 is identified as Physalin B.

**Table 2:** ^1^H NMR (400 MHz), ^13^C NMR and DEPT (100 MHz), H–H COSY and HMBC spectroscopic data for Compound 2 in CD_3_OD

position	*δ*H (mult, *J* in HZ)	*δ*C (DEPT)	H–H COSY	HMBC
1		206.39		
2	5.71 (dd, J = 10.02, 2.81)	126.98 (CH)	H-3, H-4	C-1, C-3, C-4, C-10
3	6.80 (ddd, J = 9.83, 4.70, 2.33)	146.73 (CH)	H-2, H-4	C-1, C-2, C-5
4	3.30 (m) 2.78 (dd, J = 21.65, 5.06)	33.06 (CH_2_)	H-2, H3	C-2, C-3, C-5, C-10
5		135.34 (C)		
6	5.55 (brd)	124.79 (CH)		C-4, C-7, C-10
7	1.90 (m) 2.18 (m)	25.28 (CH_2_)	H-8	C-7, C-9, C-10
8	1.82 (m)	35.78 (CH)	H-7	
9		33.82 (CH)		
10		50.73 (C)		
11		24.38 (CH_2_)		
12		28.82 (CH_2_)		
13		78.55 (C)		
14		103.52 (C)		
15		211.15 (C)		
16		55.81 (CH)		
17		87.51 (C)		
18		172.71 (C)		
19	1.30 (s)	19.61 (CH_3_)		
20	1.77 (s)	82.33 (C)		
21		19.37 (CH_3_)		
22	4.19 (m)	70.16 (CH)		C-20, C-23, C-24
23		31.63 (CH_2_)		
24		33.70 (C)		
25		48.50 (CH)		
26		167.84		
27	3.56 (m) 4.68 (m)	60.84 (CH_2_)		C-25, C-26
28	1.15 (s)	12.89 (CH_3_)		

^1^H NMR (400 MHz); ^13^CNMR (100 MHz); *J*-value is expresses in Hz between parentheses

#### Compound 3

The ^13^C NMR data of Compound 3 (Table 3) showed 28 carbon signals characteristic of C-28 steroidal lactone built on the ergostane framework. ^1^H NMR data of the compound (Table 3) supported the evidence. The presence of a 5*β*, 6*β*-epoxy-3,4 *β*-dihydroxy-1-one unit in rings A and B was deduced from the signals at δ 2.87 (2H, d, m), 4.16 (1H, m), 3.90 (1H, d, J = 7.27) and 1.52 (1H, m) and 1.89 (1H, brs) assigned to H-2, H-3, H-4 and H-7, respectively. The deduced pattern of rings A and B was finally confirmed by the carbon signals at δ ppm 210.14 (C-1). The hydroxylation at C-3 and C-4 was concluded from the downfield shift of their carbons at δ ppm 77.83 and 78.69 respectively. The allocation of C-5 and C-6 at δ ppm 72.97 and 63.52 respectively was the final confirmation of the presence of 5*β*, 6*β*-epoxy moiety. The δ-lactone side chain became evident from the characteristic signals at δ ppm 1.73 and 1.62 each integrated for 3H (two olefinic methyls, H-27, H-28 respectively) and a multiplet at δ ppm 4.62 (H-22). Moreover, the ^13^C NMR data displayed one carbonyl carbon (δ C 166.78), and two olefinic carbons (δ C 154.48 and 119.9) characteristic for the δ-lactone side chain. The downfield shift appearances of H-21 methyl at δ ppm 1.24 as a singlet signal with the absence of H-20; indicate the presence of an oxygen function at C-20. The presence of a γ-lactone carbonyl deduced from the presence of a carbonyl signal at δ ppm 175.26; together with the absence of 18-methyl in ^13^C NMR data suggest the presence of γ-lactone like that of the withaphysalins [36]. The presence of a hydroxyl group at C-14 was deduced from the downfield shift at δ ppm 87.68. The positive ESI–MS spectrum of Compound 3 gave a final confirmation of the structure that showed a molecular ion peak at *m/z* 517.24 [M + H]^+^, corresponding to the molecular weight 516.24. According to the above-mentioned data and by comparison with previously reported data of related compounds [37–39]. Compound 3 is identified as 3α, 14 *β*-dihydroxy withaphysalin N.

**Table 3:** ^1^H NMR (400 MHz), ^13^C NMR (100 MHz) spectroscopic data for Compound 3 in CD_3_OD

position	*δ*_H_ (mult, *J* in HZ)	*δ*_C_
1		210.14
2	2.87 (2H, d, m)	42.03
3	4.16 (1H, m)	77.83
4	3.90 (1H, d, J = 7.27)	78.69
5		72.97
6	Masked by solvent peak	63.52
7	1.52 (1H, m) 1.89 (1H, brs)	26.21
8		33.72
9	1.35 (1H, t, J = 9,98)	29.46
10		54.43
11		20.96
12		35.06
13		60.35
14		87.68
15		36.46
16		25.55
17		54.55
18		175.26
19	0.95 (3H, S)	15.71
20		82.53
21	1.24 (3H, S)	20.43
22	4.62 (1H, m)	81.70
23		32.27
24		154.48
25		119.94
26		166.78
27	1.72 (3H, s)	11.99
28	1.62 (3H, s)	19.70

^1^H NMR (400 MHz); ^13^CNMR (100 MHz); *J*-value is expresses in Hz between parentheses

### Evaluation of the nephroprotective effect in CdCl_2_-induced nephrotoxicity in rats

#### Acute toxicity study

At the maximum tested dose of AME and Bu.F. of *P. peruviana* L. (5 g/kg), there was no noticeable change in the behavior or mortality of mice. Therefore, doses of 500 and 1000 mg/kg in the current study were safe.

#### Effect of AME and Bu.F. of *P. peruviana* L. calyx on body weight and kidney weight

In all treated groups, standard as well as the control group, there was a non-significant increase between the initial and final body weight. On the other hand, the Cd-treated group showed a non-significant decrease between the initial and final body weight. The kidney weight of the Cd-treated group was significantly increased as compared to the control group (*p* < 0.05), while the standard and all treated groups showed a significant reduction in the kidney weight as compared to the Cd-treated group (*p* < 0.05) (Table 4).

**Table 4:** Effect of *P. peruviana* L. calyces extract and butanol fraction on body weight and kidney weight in Cd-induced nephrotoxicity in rats

Groups and doses (mg/kg)	Body weight (g)		Kidney weight (g)
	Initial	Final	
**Control**	198.00 ± 5.31	209.85 ± 6.67	1.00 ± 0.02
**CdCl**_**2**_** 3**	209.71 ± 6.44	197.85 ± 6.58	1.15 ± 0.01^a^
**AME 500**	200.14 ± 4.98	203.57 ± 5.40	0.99 ± 0.02^b^
**AME 1000**	198.85 ± 5.28	204.28 ± 4.61	0.96 ± 0.02^b^
**Bu.F. 500**	196.71 ± 4.82	201.28 ± 4.52	1.00 ± 0.02^b^
**Bu.F. 1000**	193.57 ± 4.11	198.00 ± 3.79	0.99 ± 0.01^b^
**Silymarin 150**	193.71 ± 1.97	202.14 ± 2.23	0.01^b^

Data presented as: Mean ± SE (*n* = 6)

*CdCl*_*2*_ Cadmium chloride, *AME* Aqueous Methanolic Extract, *Bu.F* Butanol Fraction

a Significant from control group at *P* < 0.05

b Significant from CdCl_2_ group at *P* < 0.05

#### Effect of AME and Bu.F. of *P. peruviana* L. calyx on serum urea and creatinine levels

Injection of CdCl_2_ in rats showed a highly significant level of serum urea and creatinine as compared to that of the control group (*p* < 0.05), revealing acute renal dysfunction. Pretreatment with AME 500 mg/kg did not significantly decrease the level of urea or creatinine when compared to the CdCl_2_ group, while the standard and all other treated groups showed a significant reduction as compared to the CdCl_2_ group (*p* < 0.05). The dose of Bu.F. 1000 mg/kg showed the best Renoprotective effect where it was non-significant from the control group (Table 5).

**Table 5:** Effect of *P. peruviana* L. calyces extract and butanol fraction on serum urea and creatinine levels in Cd-induced nephrotoxicity in rats

Groups and doses (mg/kg)	Serum urea (mg/dL)	Serum creatinine (mg/dL)
Control	5.18 ± 0.30	0.57 ± 0.04
CdCl_2_ 3	9.65 ± 0.65 ^a c^	1.32 ± 0.09 ^a c^
AME 500	8.55 ± 0.40 ^a^	1.12 ± 0.07 ^a c^
AME 1000	7.41 ± 0.26 ^a b^	1.01 ± 0.02 ^a b^
Bu.F. 500	7.15 ± 0.30 ^a b^	0.86 ± 0.02 ^a b^
Bu.F. 1000	6.05 ± 0.43 ^b^	0.70 ± 0.03 ^b^
Silymarin 150	6.90 ± 0.30 ^b^	0.84 ± 0.02 ^a b^

Data presented as: Mean ± SE (*n* = 6)

^a^ Significant from control group at *P* < 0.05

^b^ Significant from CdCl2 group at *P* < 0.05

^c^ Significant from standard group at *P* < 0.05

*CdCl2* Cadmium chloride, *AME* Aqueous Methanolic Extract, *Bu.F* Butanol Fraction

#### Effect of AME and Bu.F. of *P. peruviana* L. calyx on oxidative stress markers

**Fig. 3:**
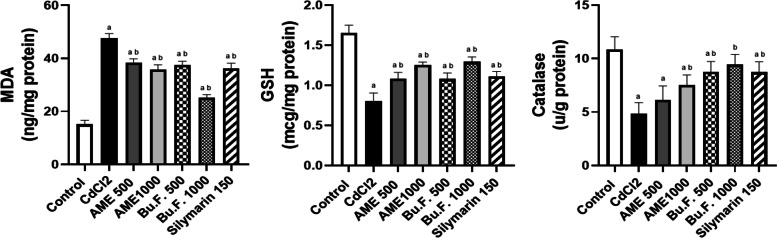
Effect of AME and Bu.F. of *P. peruviana* L. calyx on oxidative stress markers in Cd-induced nephrotoxicity in rats. Data presented as Mean ± SE (*n* = 6); a: significant from the control group at *P* < 0.05; b: significant from the CdCl_2_ group at *P* < 0.05; CdCl_2_: c: significant from the standard group at *P* < 0.05; Cadmium chloride, AME: Aqueous Methanolic Extract, Bu.F: Butanol Fraction

To evaluate whether the protective effect of *P. peruviana L*. was due to its antioxidant property, the MDA and GSH levels as well as the CAT activity in the kidney tissue homogenate were estimated. Injection with CdCl_2_ induced oxidative stress in the kidney, where Cd-intoxicated rats had significantly elevated levels of MDA and decreased levels of GSH, and CAT compared to the control group (*p* < 0.05). Moreover, treatment with standard, AME (500 and 1000 mg/kg) and Bu.F. (500 and 1000 mg/kg) significantly reduced MDA and significantly increased GSH levels as compared to the CdCl_2_ group (*p* < 0.05). In addition, the CAT activity was significantly (*p* < 0.05) increased in standard, and all treated groups expect AME (500 mg/kg) which showed a non-significant increase when compared to the CdCl_2_ group. Interestingly, Bu.F. (1000 mg/kg) showed a significant improvement in oxidative stress markers as compared to the standard group, and a non-significant difference in CAT activity when compared to the control group suggesting a better antioxidant effect (Fig. 3).

#### Effect of AME and Bu.F. of *P. peruviana* L. calyx on inflammatory markers

**Fig. 4:**
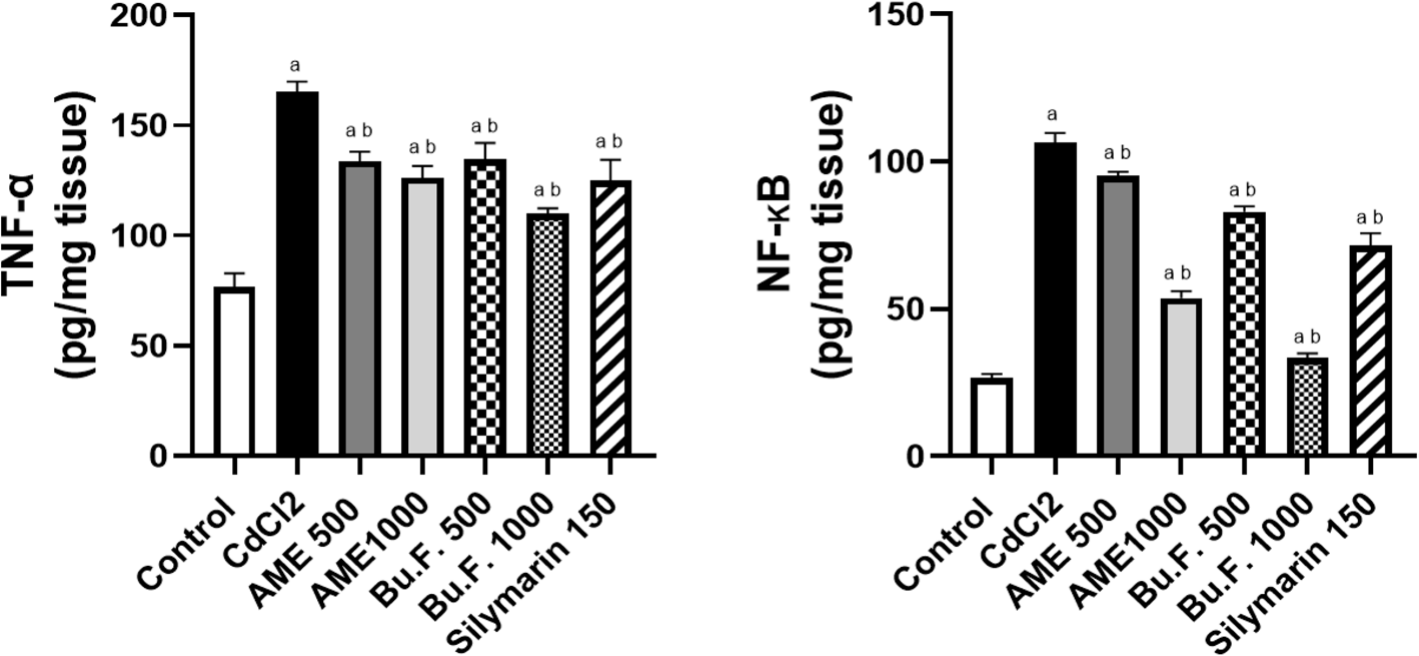
Effect of AME and Bu.F. of *P. peruviana* L. calyx on inflammatory markers in Cd-induced nephrotoxicity in rats. Data presented as Mean ± SE (*n* = 6); a: significant from the control group at *P* < 0.05; b: significant from the CdCl_2_ group at *P* < 0.05; CdCl_2_: c: significant from the standard group at *P* < 0.05; Cadmium chloride, AME: Aqueous Methanolic Extract, Bu.F: Butanol Fraction

The anti-inflammatory activity of *P. peruviana L*. was evaluated by determining the concentrations of TNF- α as a pro-inflammatory cytokine, and its transcription factor NF-kB. Cd-injected rats showed a significant increase in the concentrations of TNF- α and NF-kB as compared to the control group (*p* < 0.05). However, pretreatment with standard and all doses of the extract exhibited a significant reduction in the concentration of these inflammatory markers as compared to the Cd-treated rats (*p* < 0.05). Notably, Bu.F. (1000 mg/kg) showed a significant reduction in these inflammatory markers when compared to the standard group suggesting a stronger anti-inflammatory effect (Fig. 4).

#### Effect of AME and Bu.F. of *P. peruviana* L. calyx on kidney histology

**Fig. 5:**
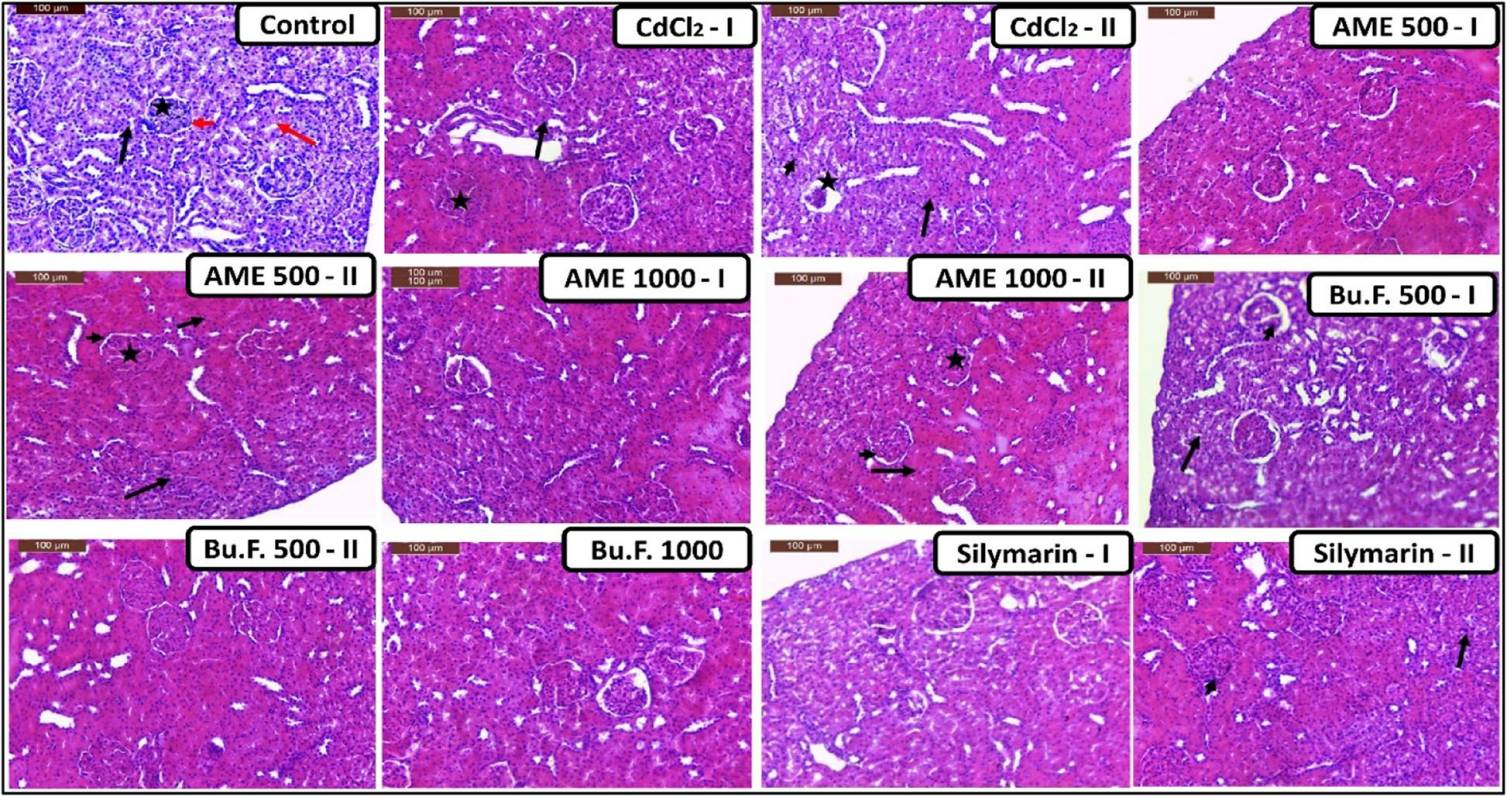
Effect of AME and Bu.F. of P. peruviana L. calyx on histopathological examinations with hematoxylin and eosin (H&E) in CdCl_2_-induced nephrotoxicity in rats: Control: Normal distal convoluted tubules (arrow), proximal convoluted tubules (red arrow), glomeruli (asterisk), and urinary spaces (short arrow); CdCl_2_-I: Inflammatory infiltration (arrow), glomerular hypercellularity (asterisk) with narrow urinary spaces (arrowhead); CdCl_2_-II: Interstitial hemorrhage (arrows), and desquamation of tubular epithelial cells (arrowhead); AME 500–1: Renal structure appeared nearly to normal form; AME 500-II: Inflammatory infiltration (arrow), glomerular hypercellularity (asterisk), with narrow urinary spaces (arrow head), and tubular interstitial hemorrhage (arrowheads); AME 1000-I: Renal structure appeared nearly to normal form; AME 1000-II: Glomerular hypercellularity (asterisk) with narrow urinary spaces (arrow head), and interstitial Hemorrhage (arrows); Bu.F. 500-I: More or less normal renal corpuscles (arrow head), and renal tubular degenerative changes (arrow); Bu.F. 500-II: Normal renal corpuscles and tubules; Bu.F. 1000: Renal corpuscles and the renal tubules appeared nearly too normal form; Silymarin-I: Renal structure appeared nearly to normal form; Silymarin-II: Glomerular congestion and hypercellularity and narrow urinary space (arrow head), and tubular degenerative changes with pyknotic nuclei (arrow) (Scale Bar 100 µm). CdCl_2_: Cadmium chloride, AME: Aqueous Methanolic Extract, Bu.F: Butanol Fraction

Histopathological examinations of the kidney from control rats showed a normal structure of glomeruli, distal and proximal convoluted tubules, and urinary spaces. However, sections of Cd-injected rats showed hemorrhage and inflammatory infiltration with fibrotic areas in the interstitial spaces. The glomeruli showed congestion and hypercellularity with narrow urinary spaces. Kidney sections from AME 500 mg/kg-treated rats showed histopathological changes nearly like the CdCl_2_ group. Treatment with standard, AME 1000, and Bu.F. 500 showed a nephroprotective activity compared to Cd-injected rats however, in some rats, glomeruli hypercellularity, narrow urinary spaces, and hemorrhagic areas in the interstitium were found. Bu.F. 1000 showed that the renal corpuscles and the renal tubules appeared nearly to normal control rats (Fig. 5).

### Molecular docking study of the isolated compounds

**Fig. 6:**
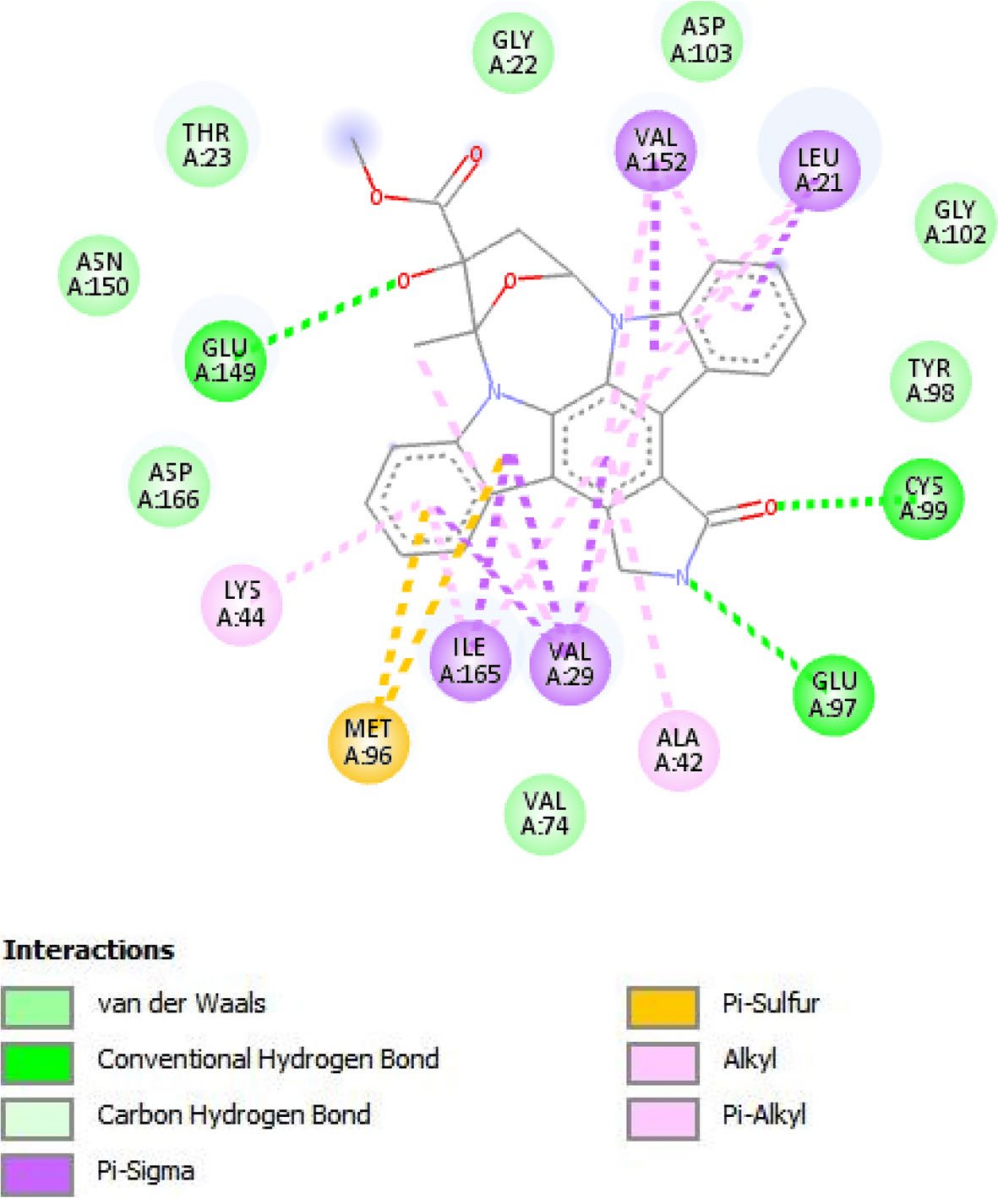
2D view of Co-crystallized Ligand with the key amino acid of 4KiK protein

A molecular docking simulation study of isolated compounds 1–3 was performed to predict the anti-inflammatory activity results. Moreover, help us to understand the binding modes and various interactions between the ligands and active sites of Human IκB kinase. The Key Amino acids for Human IκB kinase (PDB Code 4KiK) were Glutamate 149, Glutamate 97, Cysteine 99, Valine 29 and Leucine 21, as represented in (Fig. 6).

**Fig. 7:**
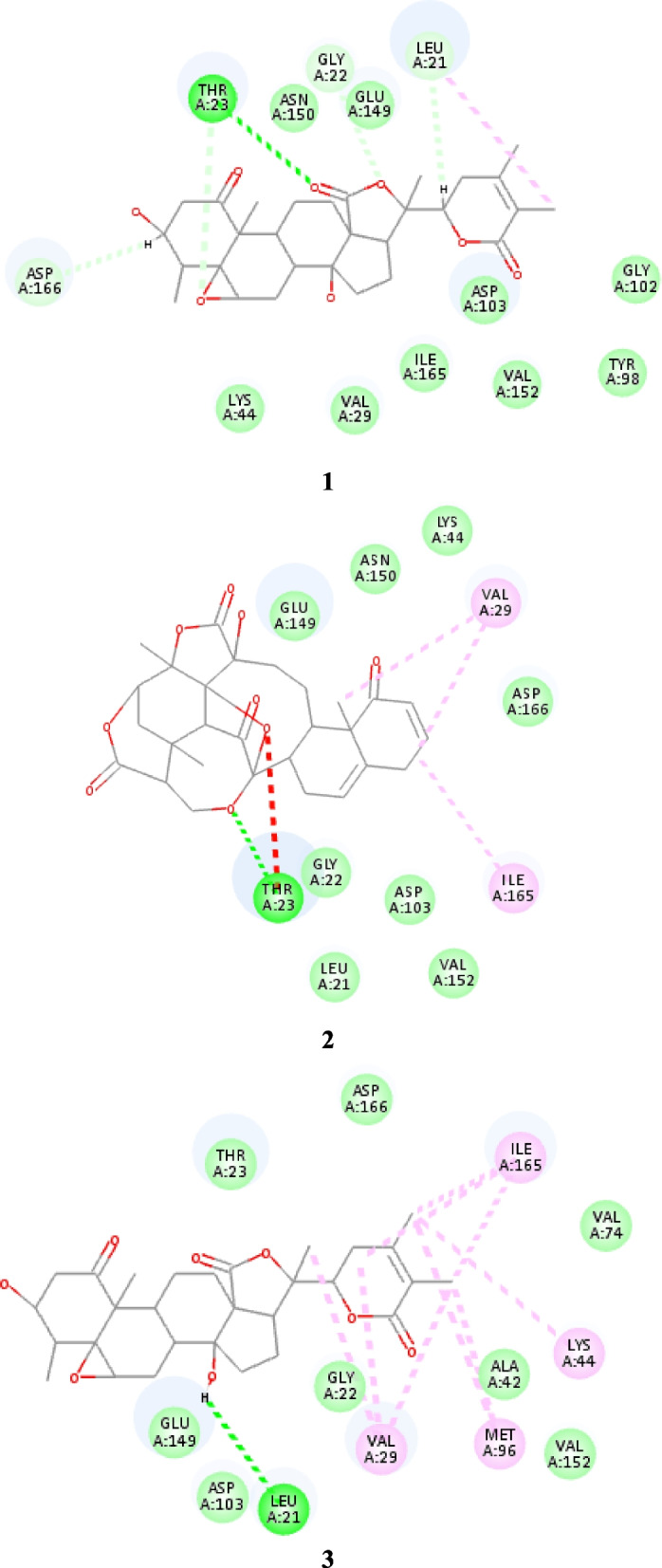
Selected key 2D docking views of Compounds 1–3

Results of the molecular docking are represented (Fig. 7). Compound 1 showed a similar orientation in the binding pocket of Human IκB kinase as the native ligand, particularly, carbon-hydrogen bond interactions towards the proton acceptors residues GLU 149, ASN 150, and LEU 21 and conventional hydrogen interactions with THR 23, GLY 22 and with alkyl interaction with ILE 165, VAL 29 and LEU 21, which accounts for the highest binding affinity toward the target protein with a free energy value of -10.6065 kcal/mol. Compound 2 displayed a binding affinity with a free energy value of -9.8216 kcal/mol, it displayed Alkyl interaction with VAL 29 (2 bonds) and ILE 165 (1 bond) through methyl and benzene groups, moreover, hydrogen bonds were observed with THR 23. Compound 3 showed several key residue interactions with Human IκB kinase beta protein summarized as conventional hydrogen interactions with THR 23, ASN 150, carbon-hydrogen bond interactions with LEU 21, GLU 149, GLY 22, and Pi-Alkyl non-covalent bond was seen between the aromatic ring and ILE 165, 2 showed a binding affinity with a free energy value of -10.401 kcal/mol.

## Discussion

Cadmium is a heavy metal that is well-known as an environmental toxin and has many toxic and hazardous impacts on human health, especially nephrotoxicity [23]. The mechanism of Cd in the pathophysiology of renal damage includes the production of reactive oxygen species (ROS) and disruption of the endogenous antioxidant system, which subsequently induces inflammatory reactions [40]. Therefore, antioxidants and anti-inflammatories may be useful therapeutic strategies against Cd-induced toxicity. Several studies investigated the role of natural products against Cd-induced toxicity [41].

Because of their relative safety, efficacy and protective effects, natural products may be used as alternative and adjuvant therapy to conventional drugs. Current pharmaceutical industries rely heavily on the discovery of secondary metabolites found in plants to aid in the development of novel drugs with potential biological activity [42]. As a result, screening medicinal plant extracts, as well as isolating and identifying their bioactive compounds are critical in the development of effective medications [43]. Based on the literature, *P. peruviana* L. is rich in withanolides [38, 44], so it was interesting to investigate in depth. Withanolides are natural steroidal lactones metabolized mainly by plants belonging to the family Solanaceae mainly the genus *Physalis*. Such secondary metabolites are excellent therapeutic agents for a variety of disorders as microbial infections, inflammations, and cancer [45, 46].

Moreover; many studies documented the antioxidant activity of withanolides [4, 47, 48]. In the current study, we aimed to investigate the possible protective effect of AME and Bu.F. of *P. peruviana* L. against Cd-induced nephrotoxicity in rats.

Intraperitoneal injection of CdCl_2_ in rats showed significant renal damage, evidenced by a significant elevation of serum urea and creatinine, these findings were in harmony with the results of [49]. In addition, Cd -injected rats showed a significant increase in the weight of the kidney due to edema caused by tubular cell damage [50], which was confirmed by the changed renal tubular structure as mentioned in the histopathological investigation.

AME and Bu.F. of *P. peruviana* L. significantly ameliorate renal dysfunction induced by Cd intoxication which was evidenced by low levels of serum urea and creatinine. CdCl_2_ induces nephrotoxicity by the generation of ROS and makes the cells more susceptible to oxidative stress like that of different pathogenesis. Free radicals disrupt the structural and functional integrity of cells by a variety of processes, including lipid peroxidation, sulfhydryl oxidation, proteolysis, and nuclear shearing as a result, this causes elevated levels of MDA and decreased levels of both GSH and CAT which are considered the defense mechanism of healthy cells through effectively scavenging these radicals (antioxidant effects). Briefly, in a healthy human or animal body, there is a balanced relationship between the generated free radicals and the antioxidant defense system. But in some pathological conditions, the cells become under oxidative stress leading to the generation of large amounts of free radicals putting a greater demand on the antioxidant defense system. Natural antioxidants act by non-enzymatic mechanisms to neutralize free radicals [51]. Pretreatment with *P. peruviana* L. calyx extracts mainly Bu. F. was able to act as natural antioxidants through decreasing MDA levels and increasing both GSH and CAT levels, revealing the nephroprotective activity of *P. peruviana* L. calyx extracts. On the other hand, CdCl_2_ caused hemorrhage and inflammatory infiltration with fibrotic areas in the interstitial spaces. The glomeruli showed congestion and hypercellularity with narrow urinary spaces that were linked with the increase in kidney weight. In agreement with [40], inflammation was also documented by the elevated levels of TNF- α which is a pro-inflammatory cytokine, and its transcription factor NF-kB. Activation of IκB kinase is responsible for phosphorylation and degradation of the inhibitory subunit IκB, rendering NF-κB free and active for inducing the transcription of several pro-inflammatory cytokines including TNF-α and IL-1β. Thus, the inactivation of IκB kinase prevents NF-κB-induced inflammatory pathways. The molecular docking study of the isolated compounds revealed a good binding affinity toward IκB kinase, which leads to the inactivation of the inflammatory pathway [52]. These findings support the In vivo study, which found that pretreatment with AME and Bu.F. of *P. peruviana* calyx caused decreasing the levels of these markers suggesting that good anti-inflammatory activity. Therefore, the anti-inflammatory activity of the extracts could be attributed to their withanolides content.

## Conclusion

Improvements in the traditional medical systems became crucial thus, investigations of ethnobotanicals became paramount to the current research interest to validate and rationalize the ethnomedicinal applications of medicinal plants. The current study reported the isolation of withanolides from *P. peruviana* L. calyx. Evaluation of the nephroprotective activity of AME and Bu.F. against cadmium chloride-induced nephrotoxicity in rats.

## Supplementary Information

**Additional file 1.**

**Additional file 2.**

**Additional file 3.**

## Data Availability

The data and figures supporting our findings can be found in the main text. NMR spectra of the isolated compounds are available as supplementary files.

## Abbreviations

AME: Aqueous methanol extract
Bu.F: *n*-Butanol fraction
AKI: Acute kidney injury
CKI: Chronic kidney injury
CAT: Catalase
GSH: Reduced glutathione
MDA: Malondialdehyde
NF-κB: Nuclear factor-kappa B
TNF- α: Tumor necrosis factor-alpha
