# Supplementary material for: Nephroprotective effect of Physalis peruviana L. calyx extract and its butanolic fraction against cadmium chloride toxicity in rats and molecular docking of isolated compounds
Source: BMC Complement Med Ther. 2023 Jan 27;23:21. doi: 10.1186/s12906-023-03845-9 (PMC9881262; doi:10.1186/s12906-023-03845-9)
Supplement: Supplementary file 1 — Additional file 1. [file 12906_2023_3845_MOESM1_ESM.pdf]

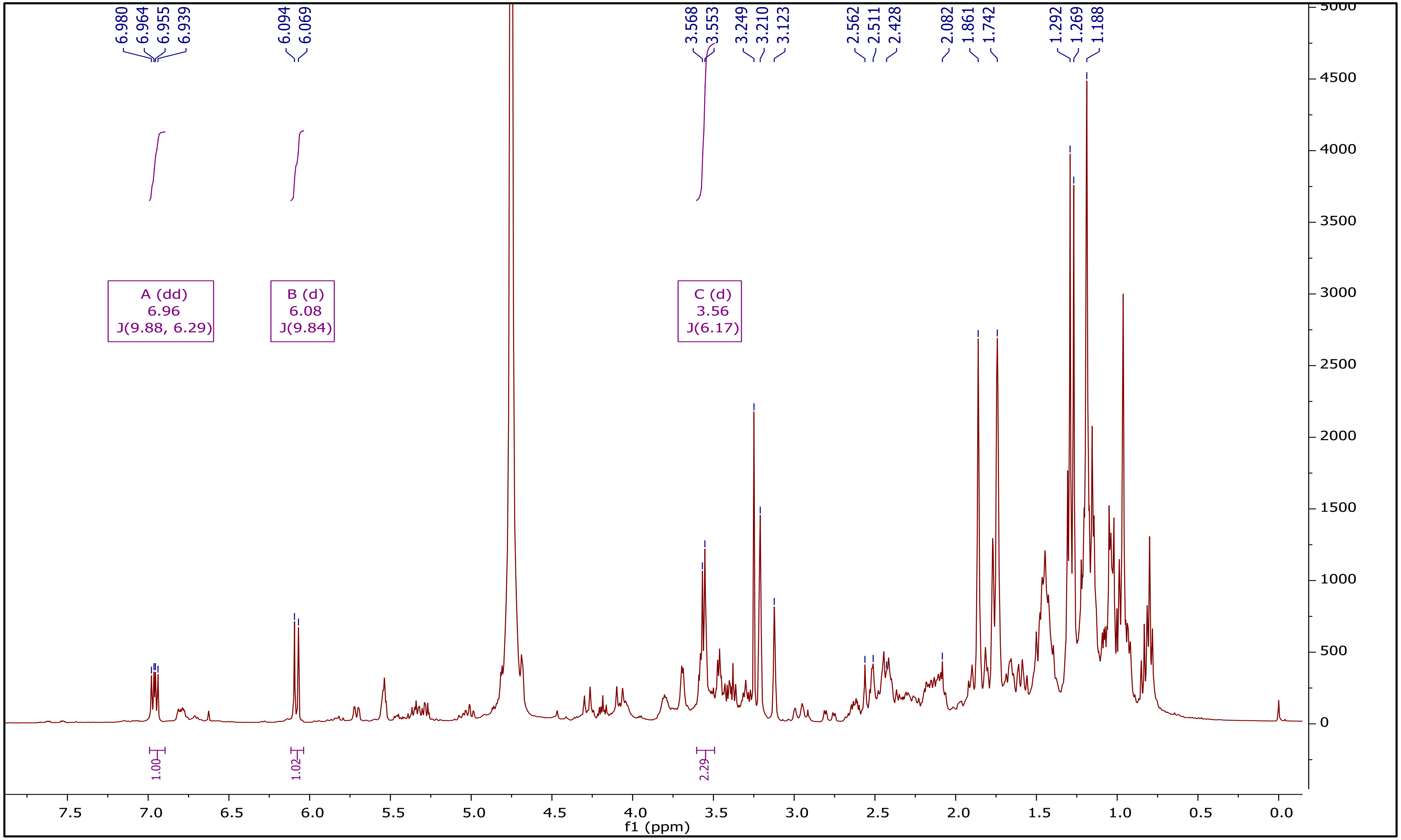

^{1}H NMR (400 MHz, CD_3OD) spectrum of compound **1**

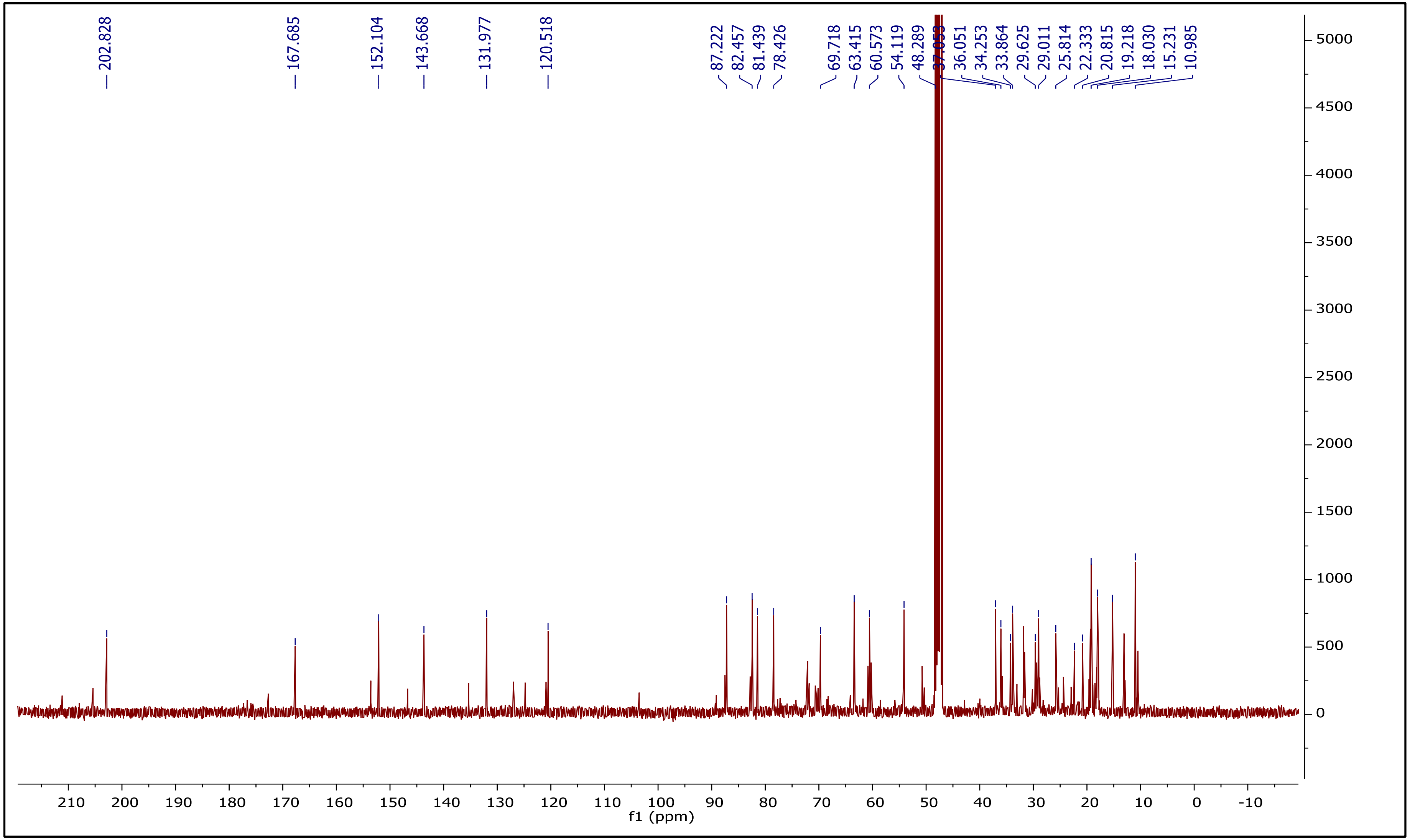

^{13}C NMR (100.40 MHz, CD_3OD), spectrum of compound **1**

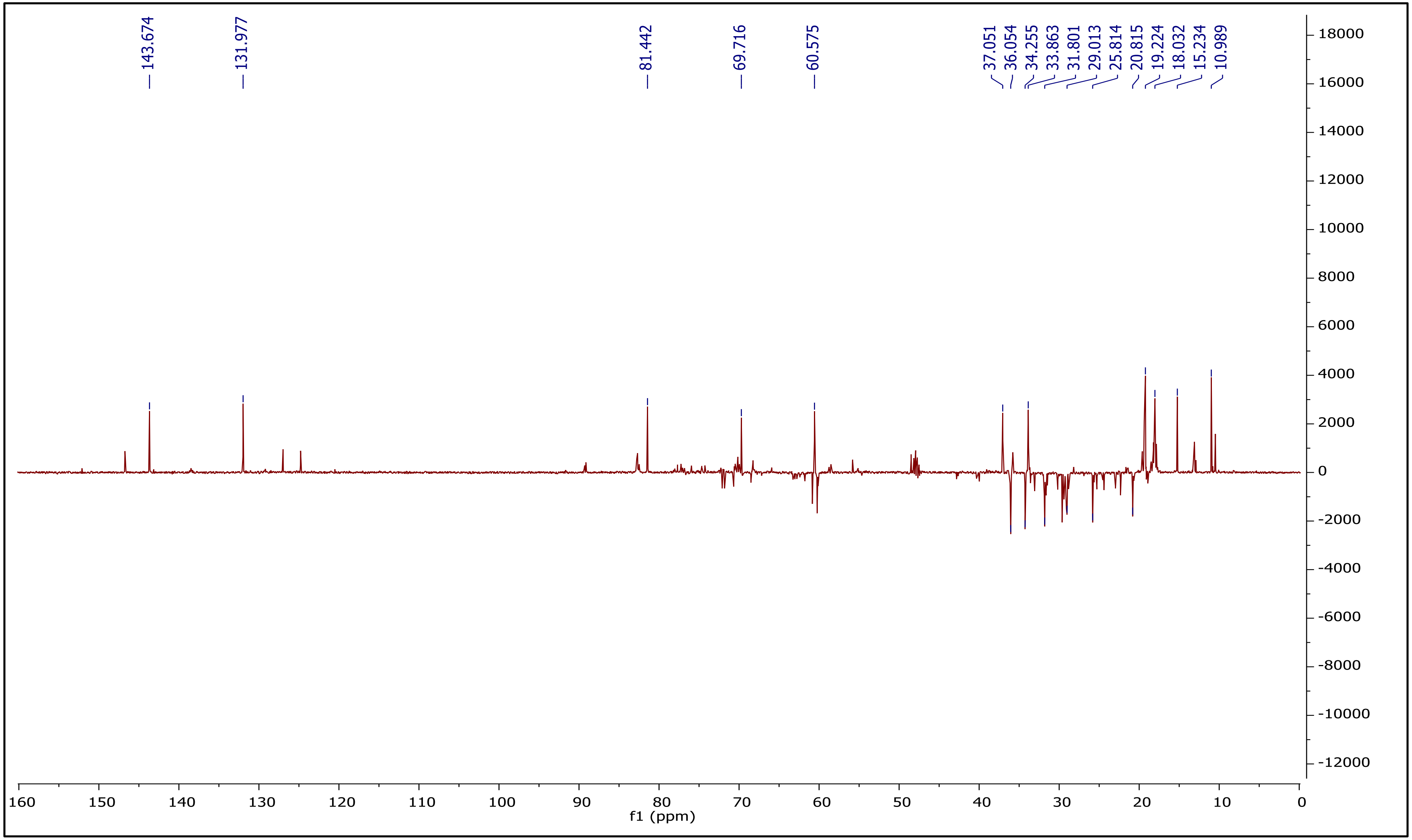

DEPT spectrum of compound **1**

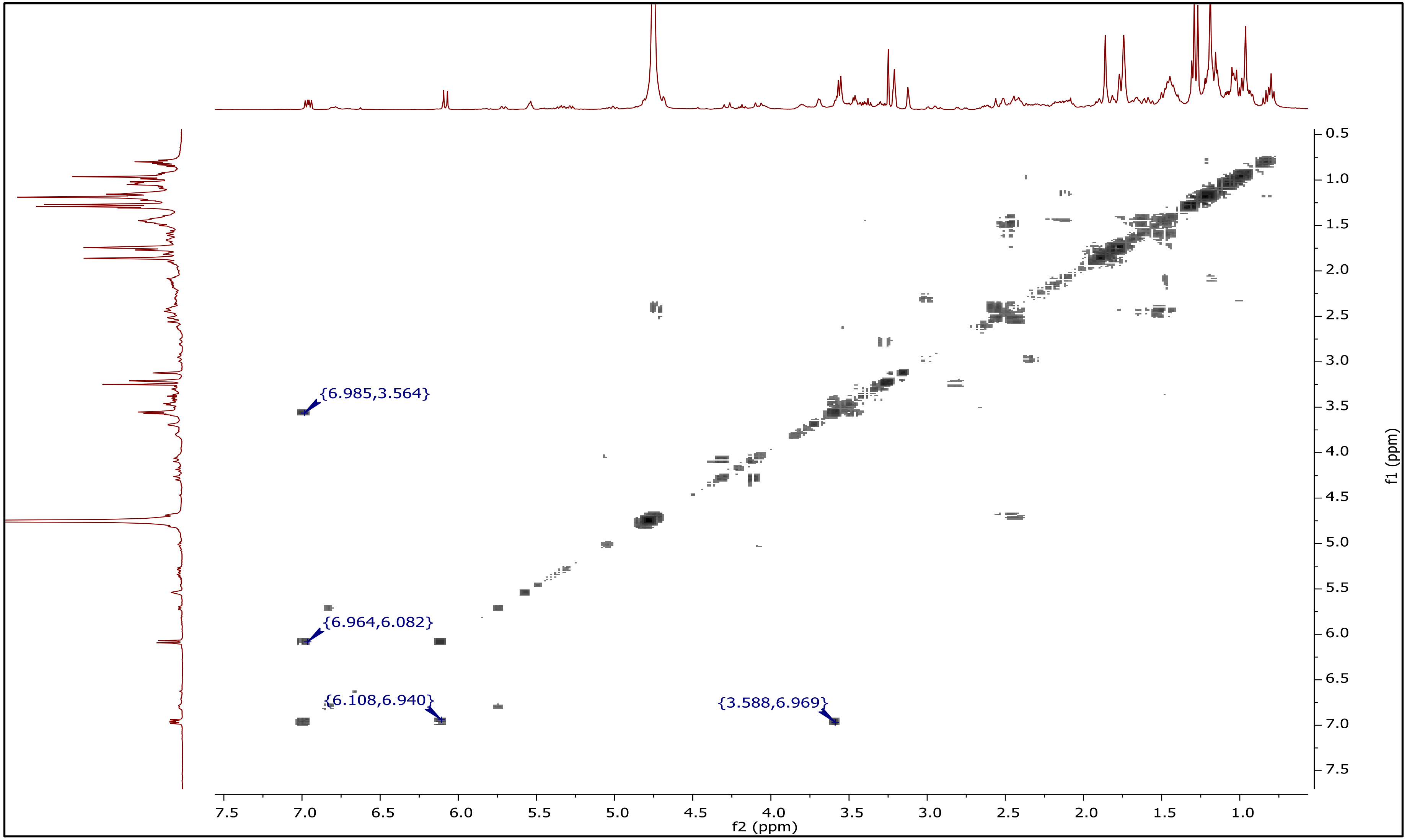

H-H COSY spectrum of compound **1**

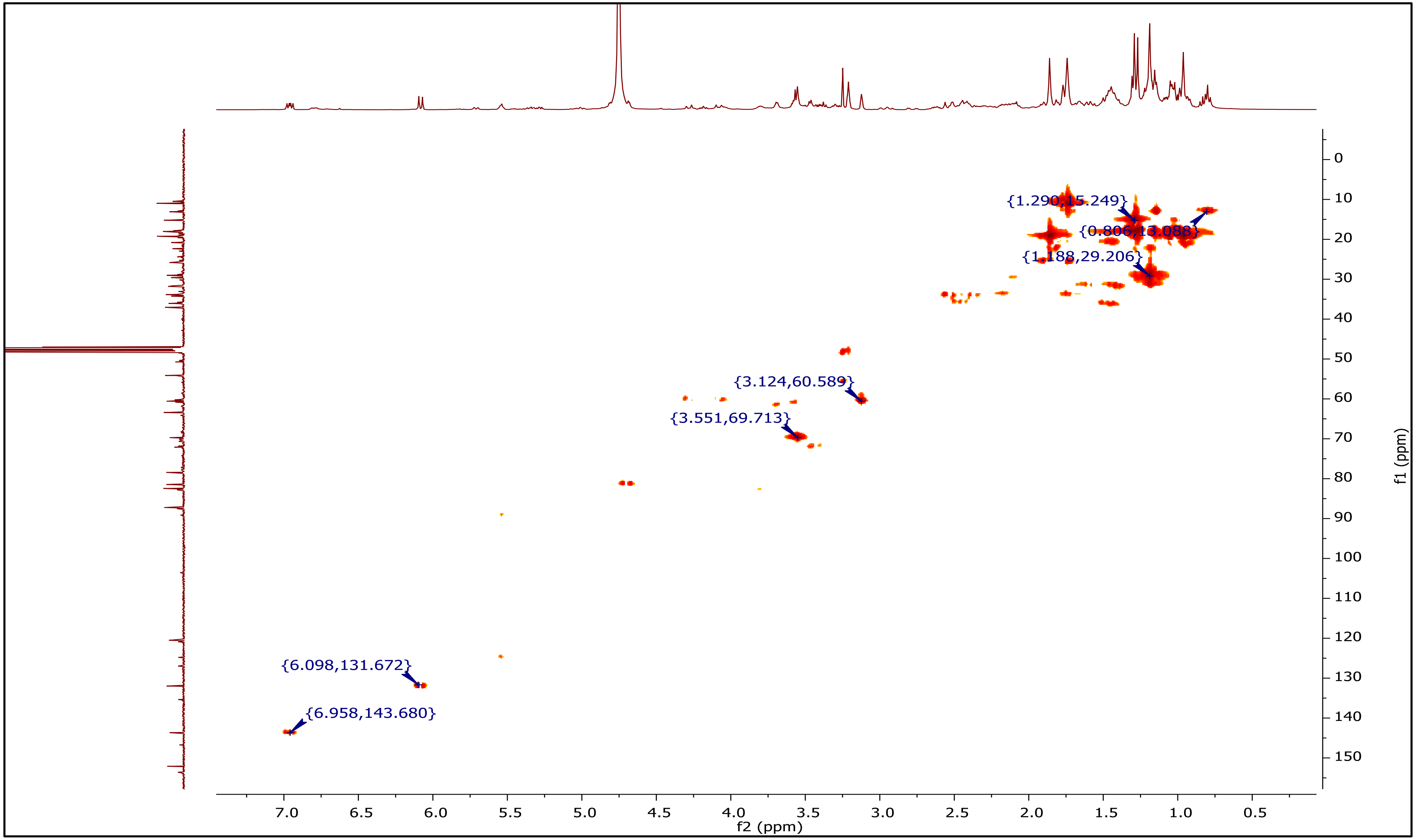

HMQC spectrum of compound **1**

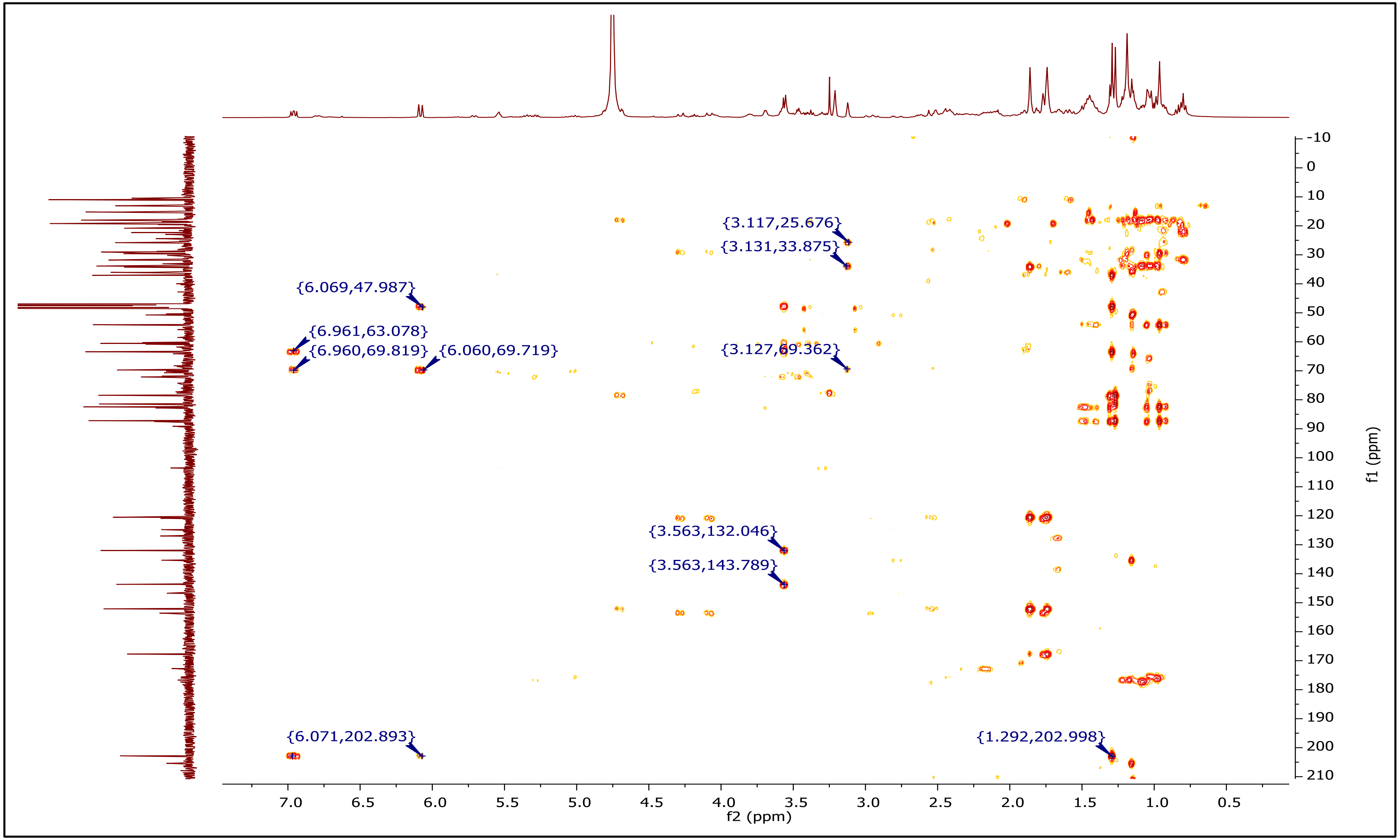

HMBC spectrum of compound **1**

Center for DRUG DISCOVERY RESEARCH and DEVELOPMENT
Openlynx Report -

Sample: 843 | Vial:1:A,2 | ID:
File:F21 72 | Date:08-Dec-2021 | Time:15:07:08
Description:EB

Printed: Mon Dec 13 13:12:23 2021

Peak ID	Time	Error PPM
28	9.12	

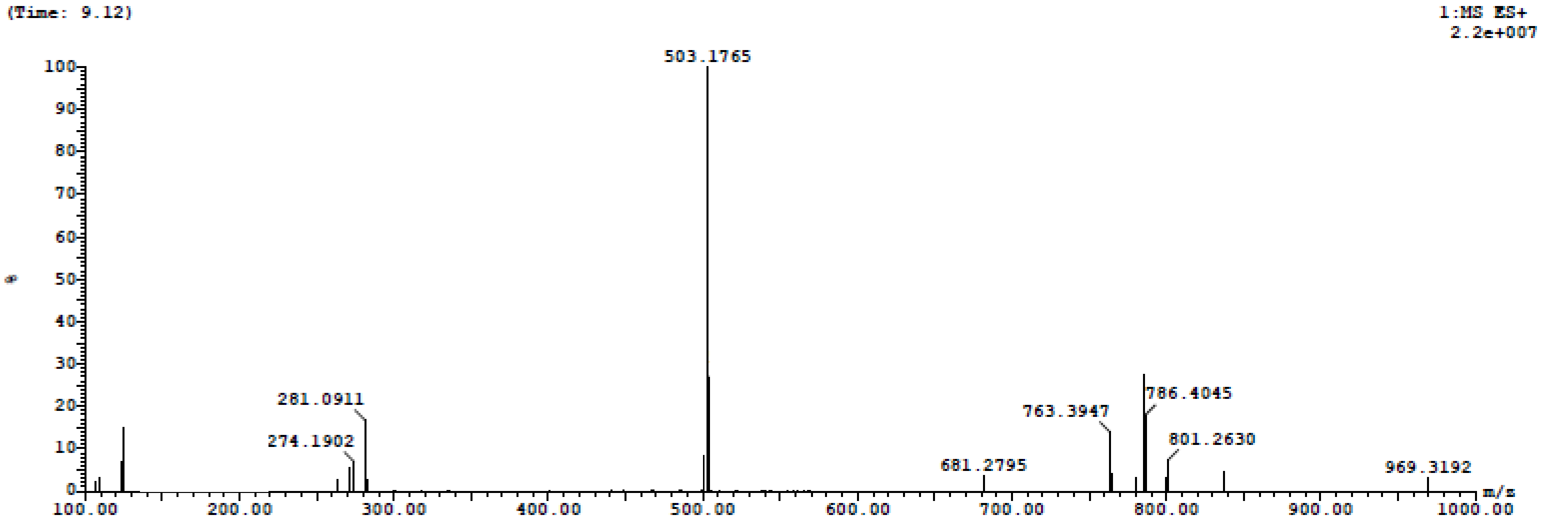

(+) ESI-MS spectrum of Compound **1**
