# Supplementary material for: Nephroprotective effect of Physalis peruviana L. calyx extract and its butanolic fraction against cadmium chloride toxicity in rats and molecular docking of isolated compounds
Source: BMC Complement Med Ther. 2023 Jan 27;23:21. doi: 10.1186/s12906-023-03845-9 (PMC9881262; doi:10.1186/s12906-023-03845-9)
Supplement: Supplementary file 2 — Additional file 2. [file 12906_2023_3845_MOESM2_ESM.pdf]

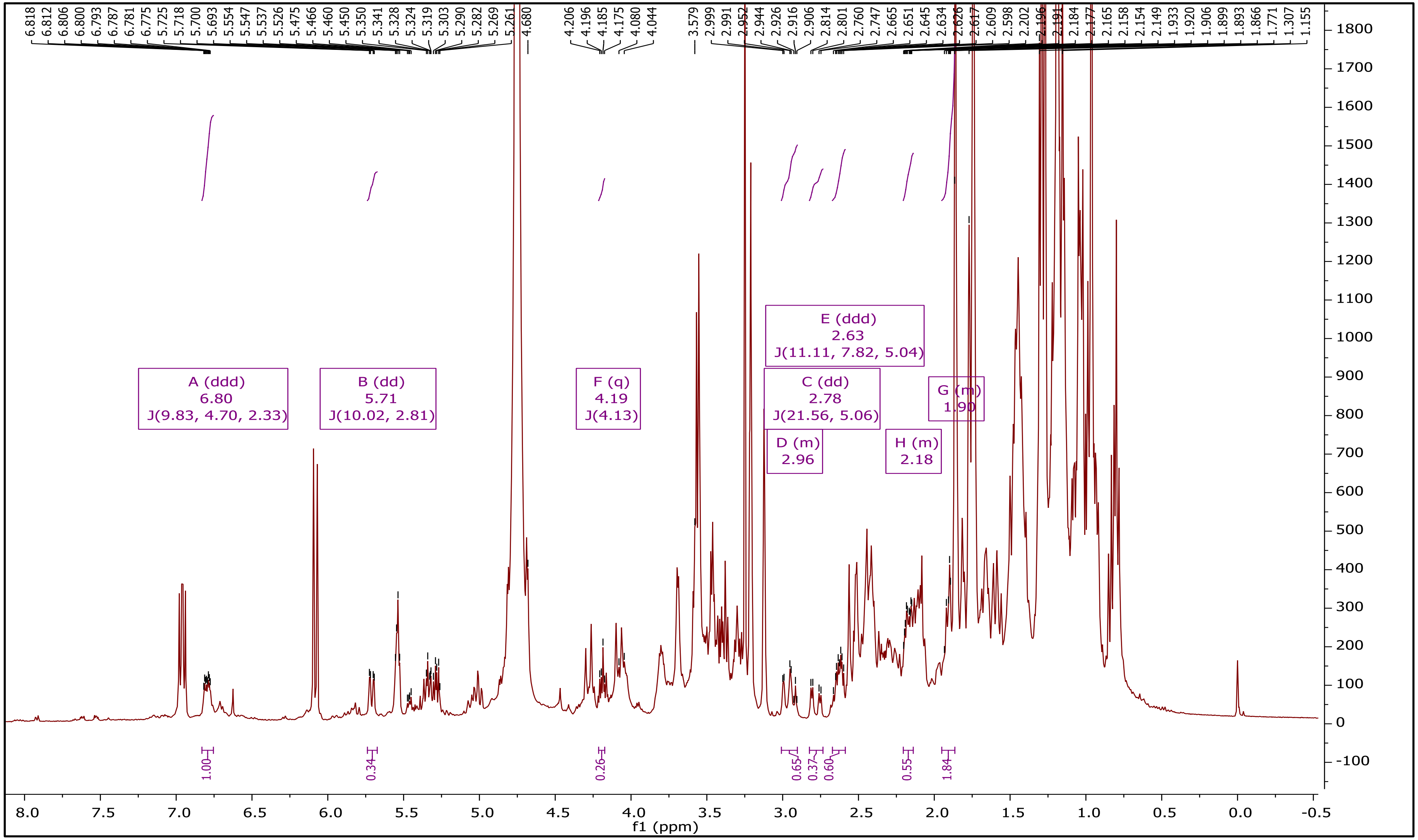

^{1}H NMR (400 MHz, CD_3OD) spectrum of compound **2**

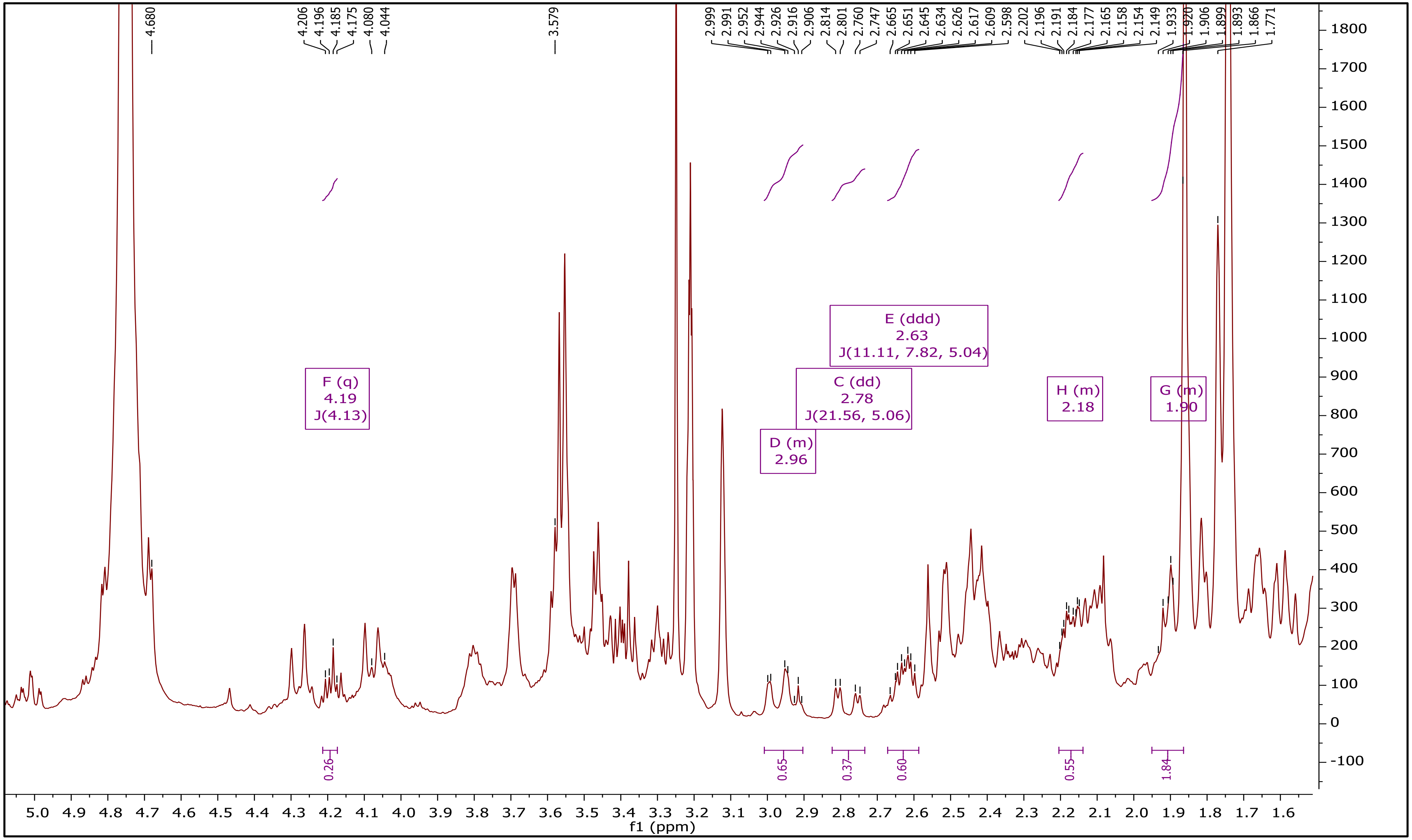

Magnification of ^{1}H NMR (400 MHz, CD_3OD) spectrum of compound **2 (δ ppm 1.5 -5)**

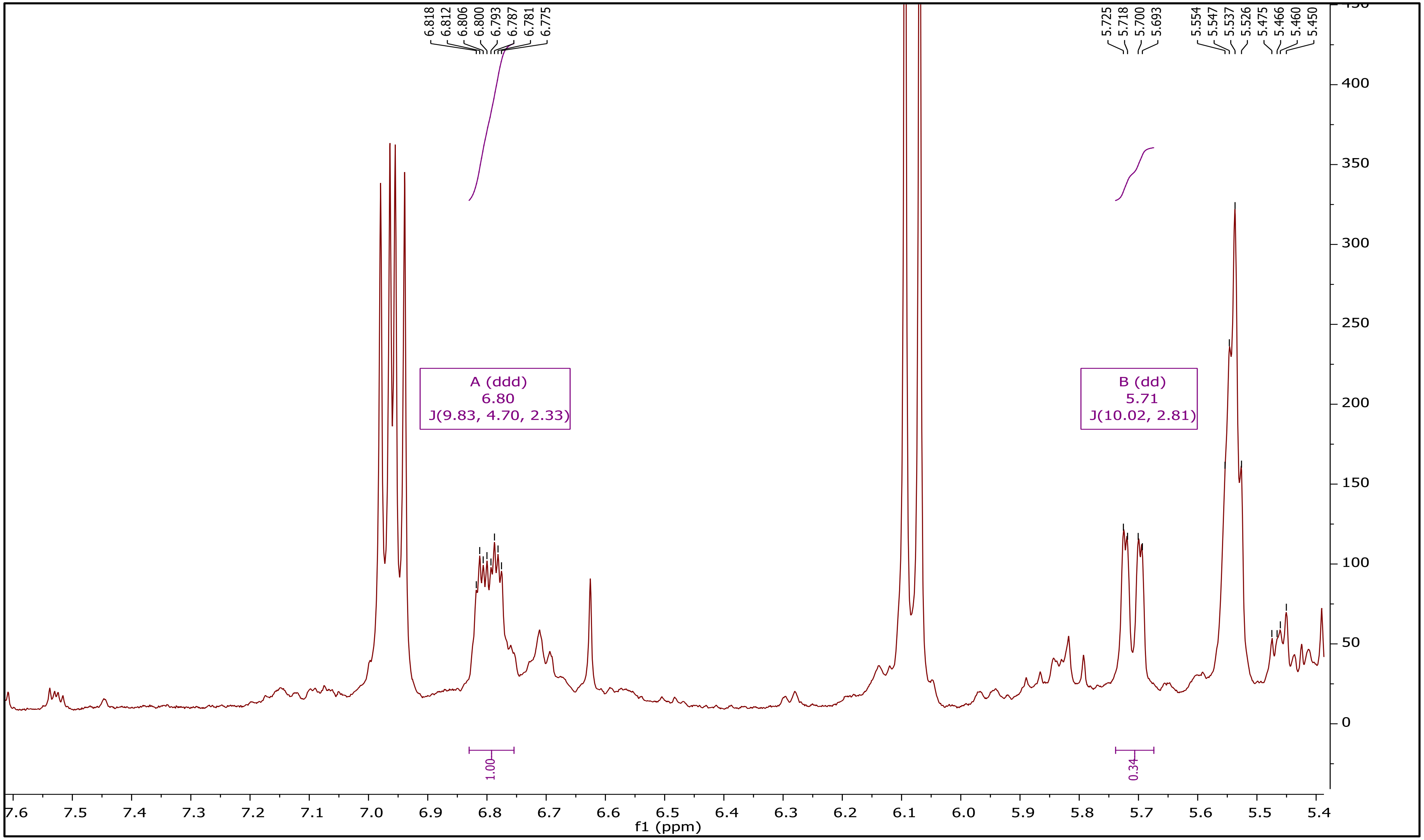

Magnification of ^{1}H NMR (400 MHz, CD_3OD) spectrum of compound **2** **(δ ppm 5.4 -7.6)**

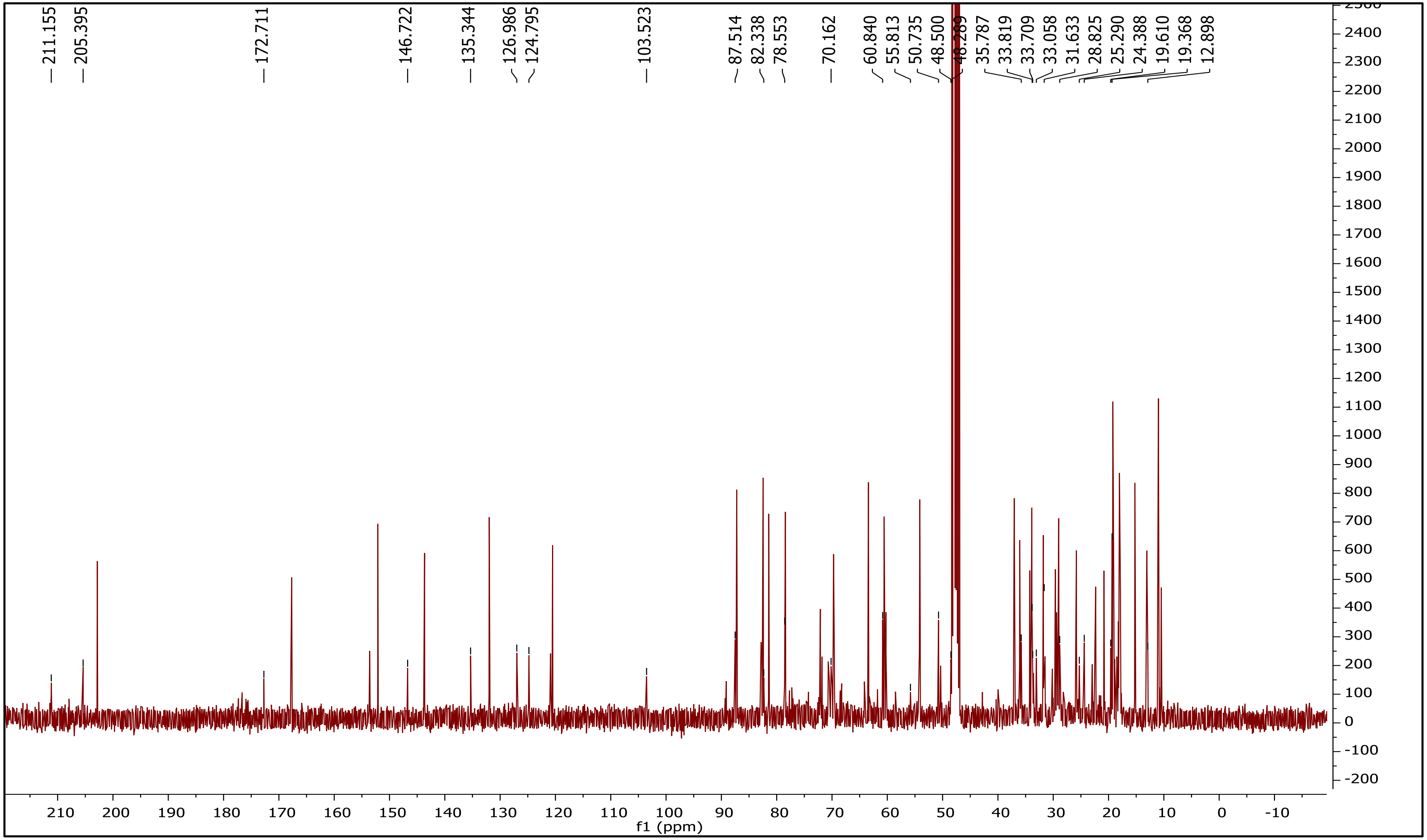

^{13}C NMR (100.40 MHz, CD_3OD), spectrum of compound **2**

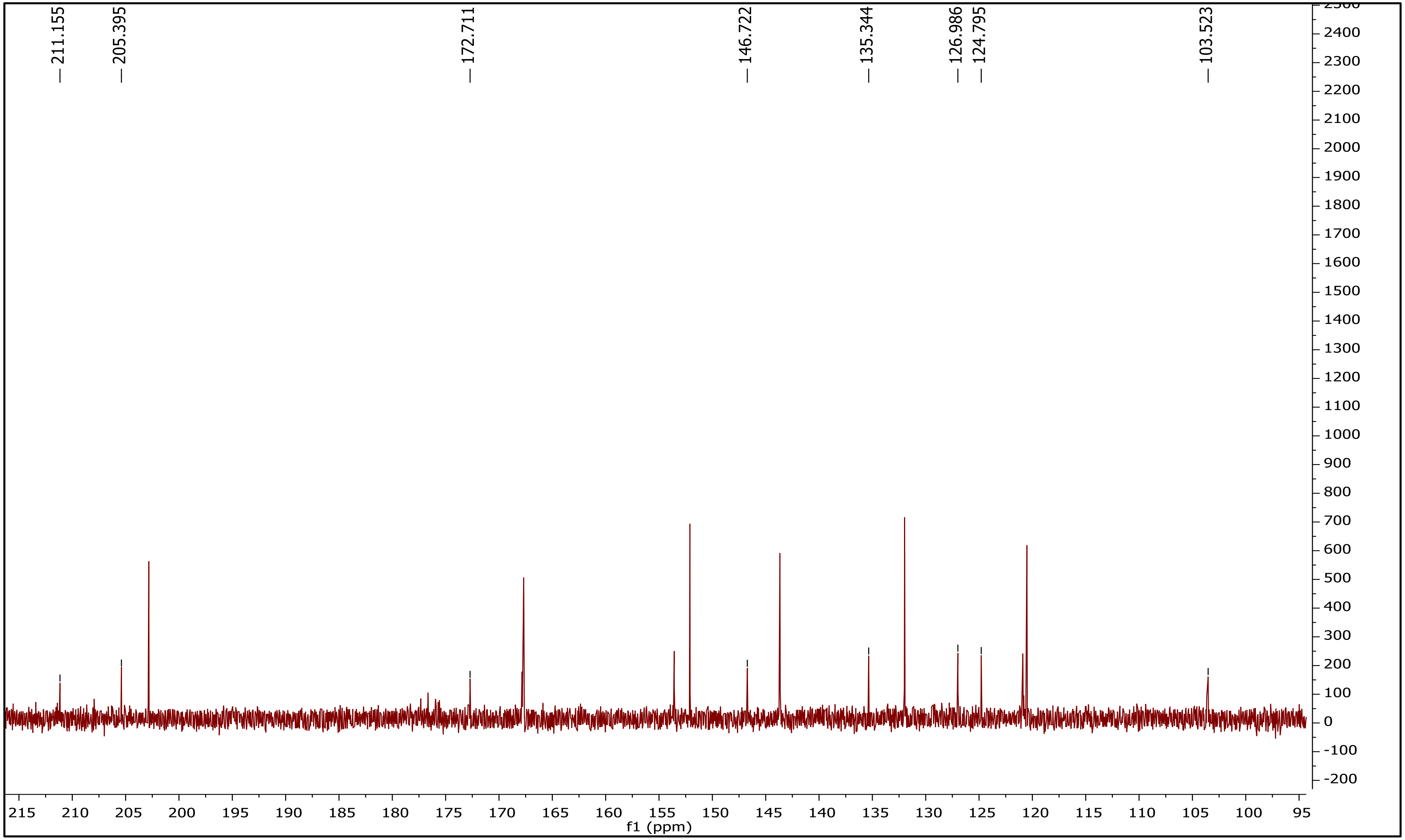

Magnification of ^{13}C NMR (100.40 MHz, CD_3OD), spectrum of compound **2** **(δ ppm 95 - 215)**

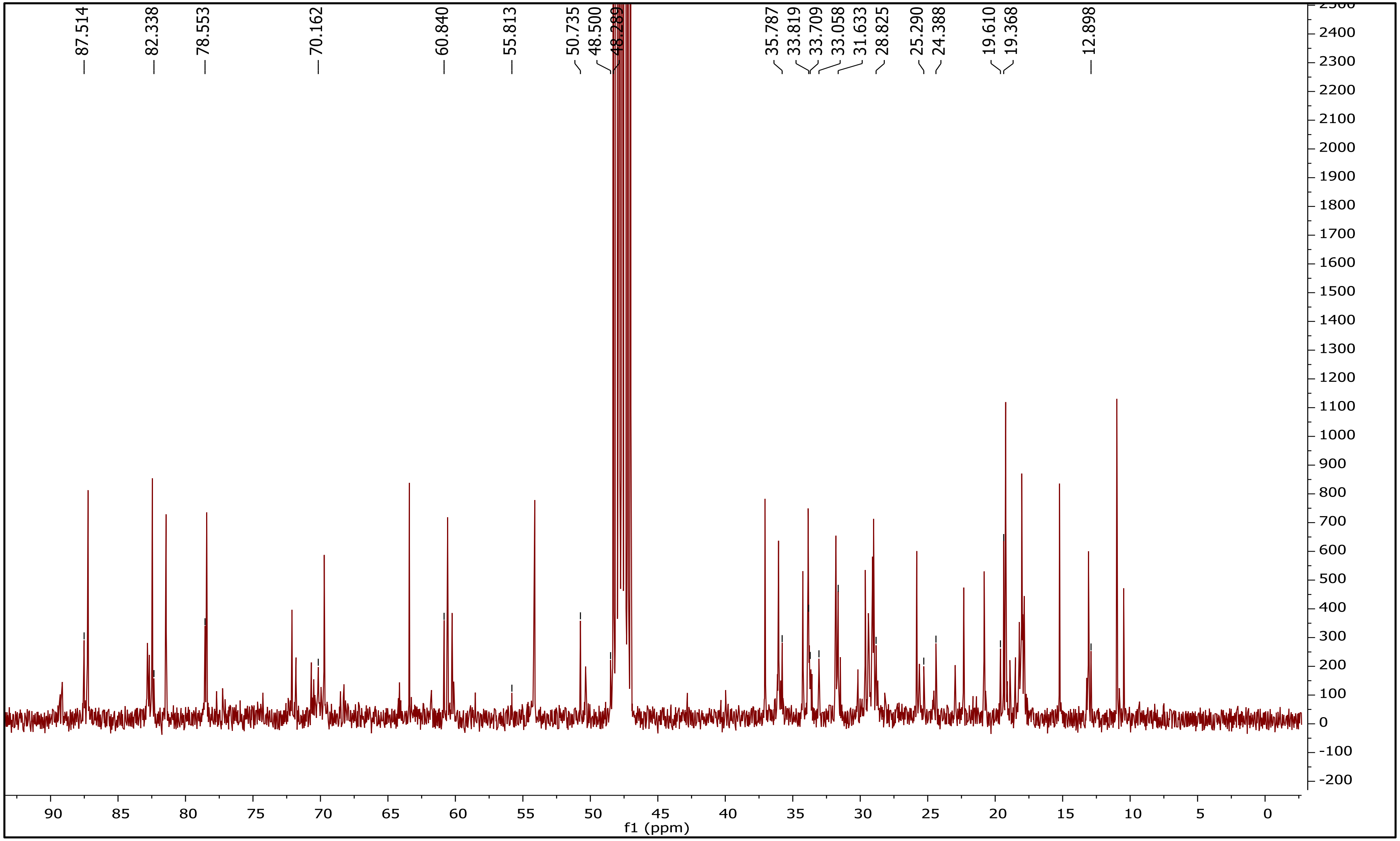

Magnification of ^{13}C NMR (100.40 MHz, CD_3OD), spectrum of compound **2** **(δ ppm 0 - 90)**

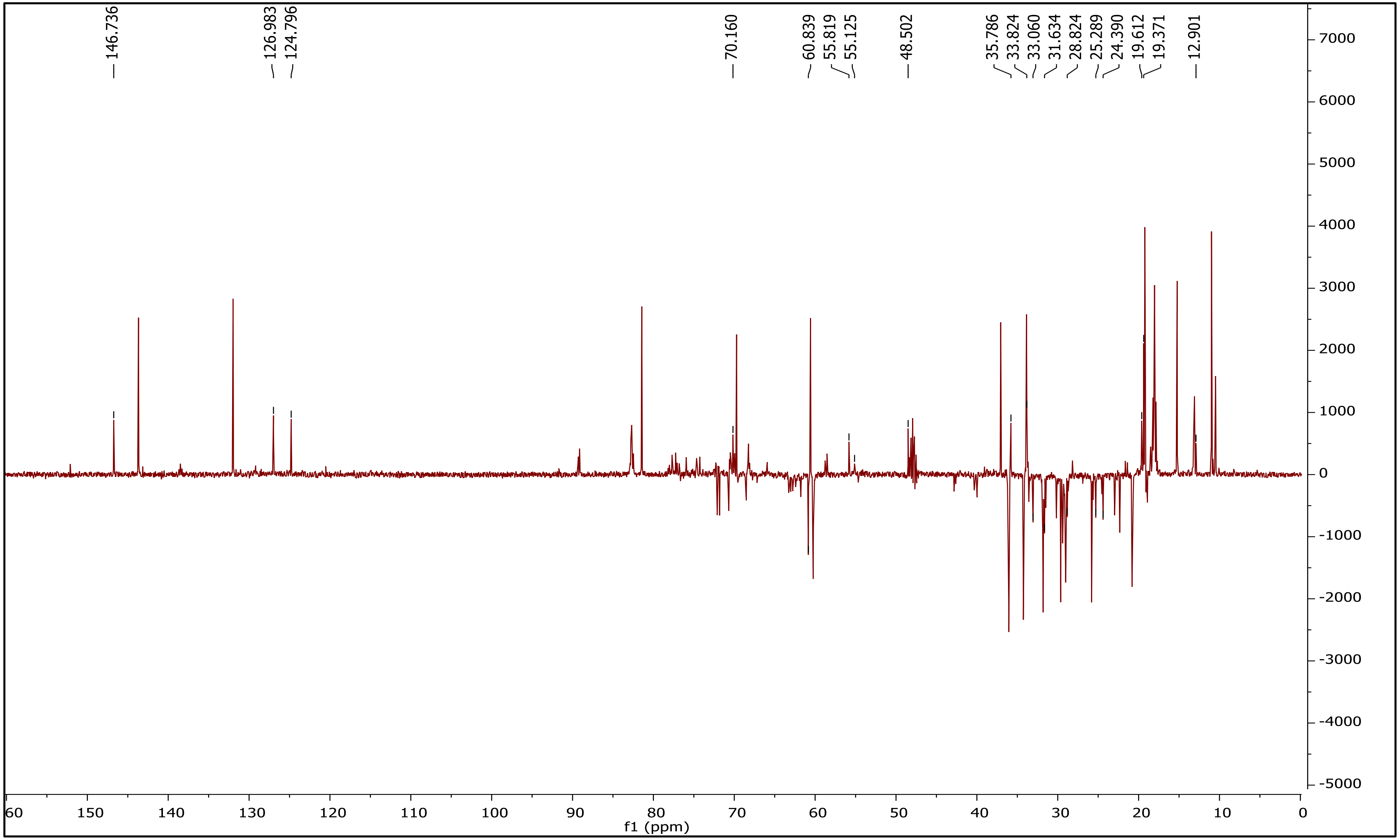

DEPT spectrum of compound **2**

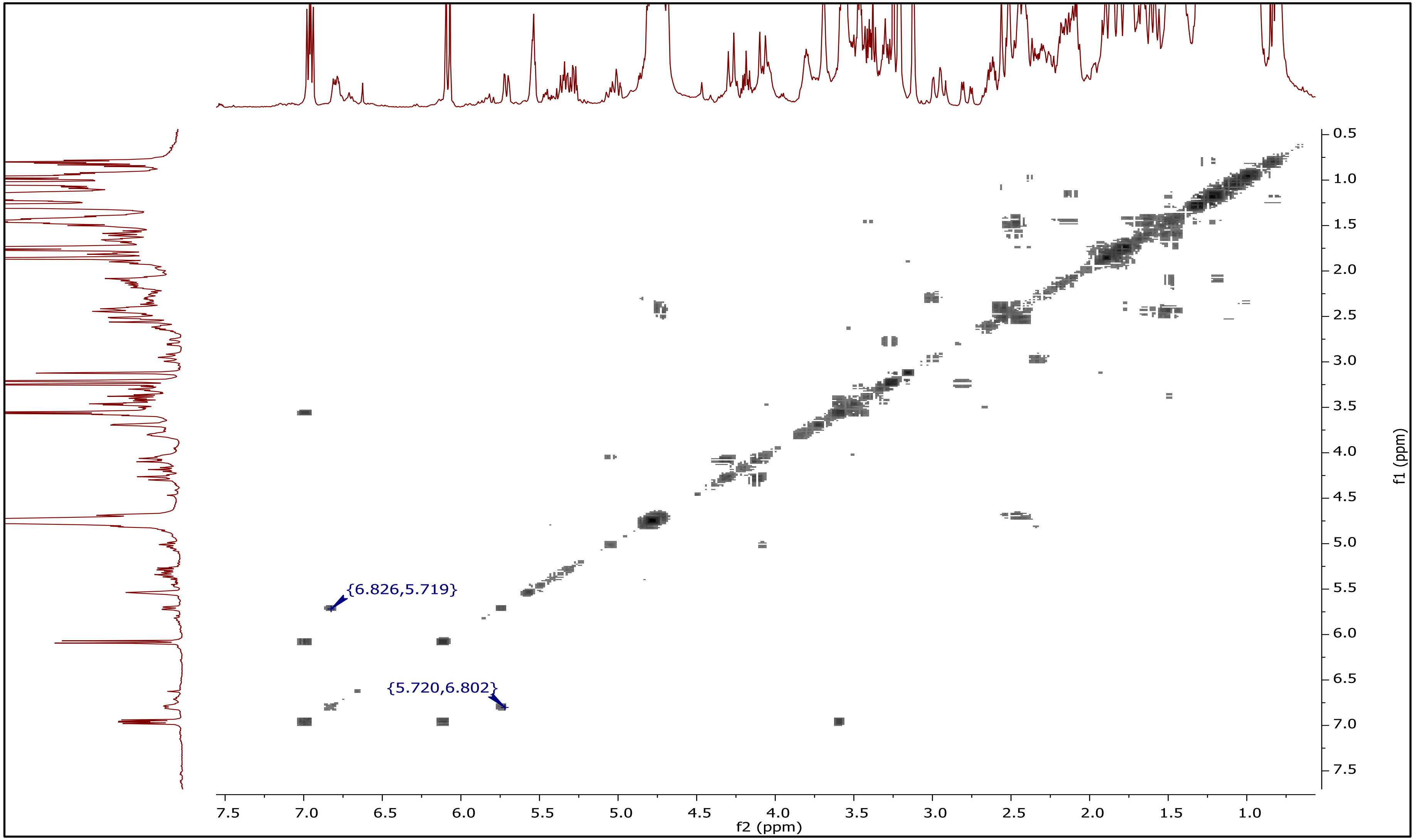

H-H COSY spectrum of compound **2**

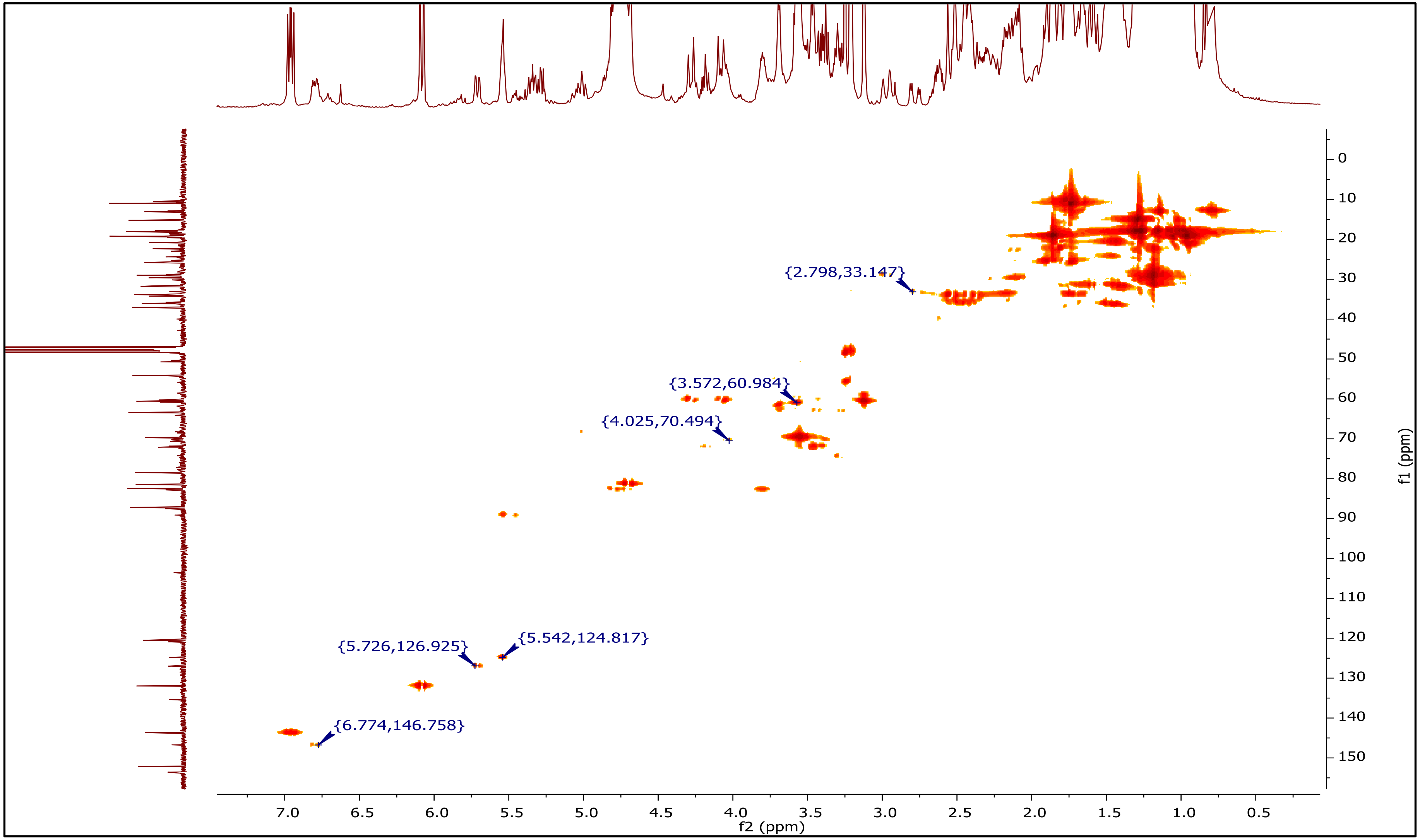

HMQC spectrum of compound **2**

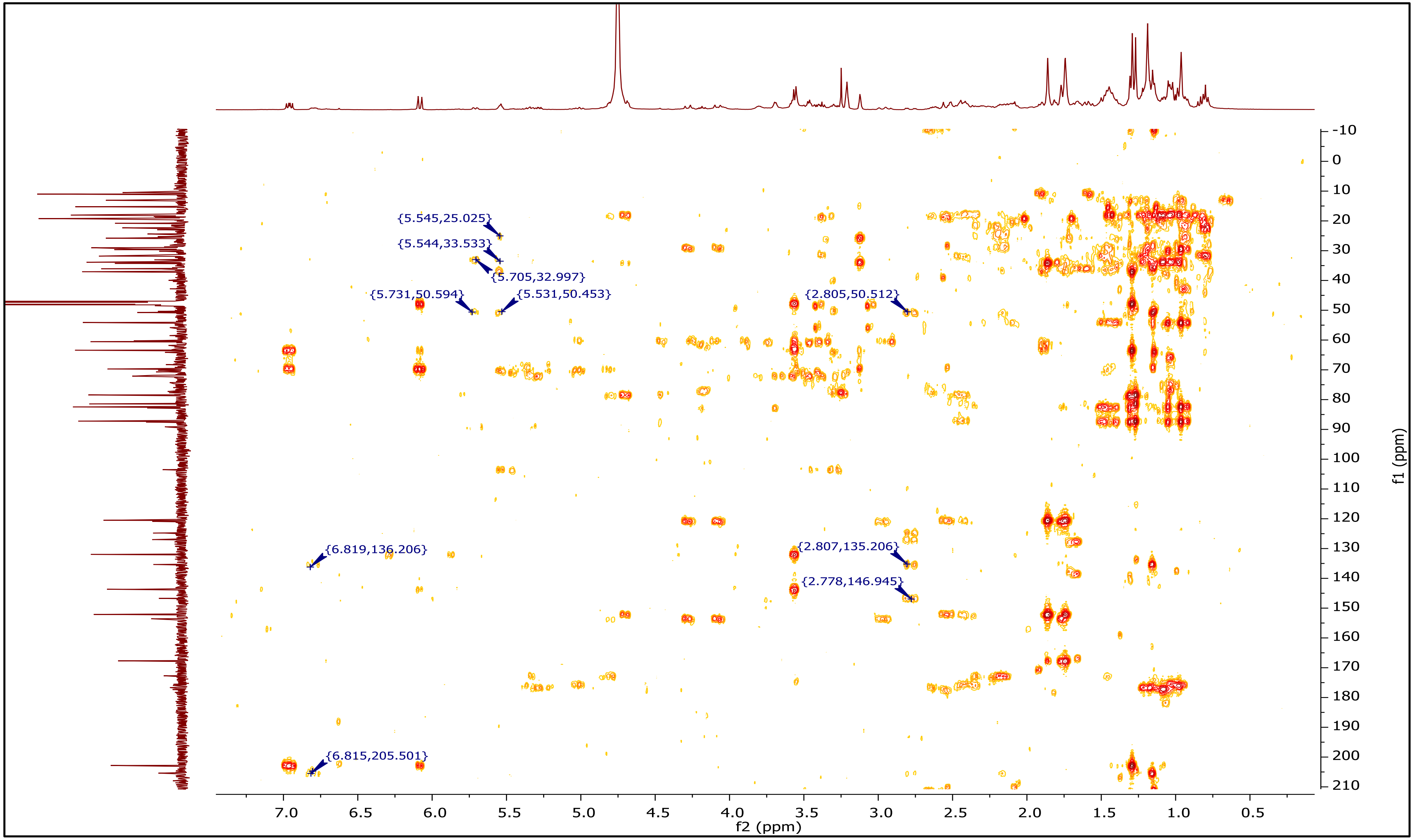

HMBC spectrum of compound **2**

Center for DRUG DISCOVERY RESEARCH and DEVELOPMENT
Openlynx Report -

Sample: 843	Vial:1:A,2	ID:
File:F21 72	Date:08-Dec-2021	Time:15:07:08
Description:EB		

Printed: Mon Dec 13 13:12:23 2021

Peak ID	Time	Error PPM
54	16.10	

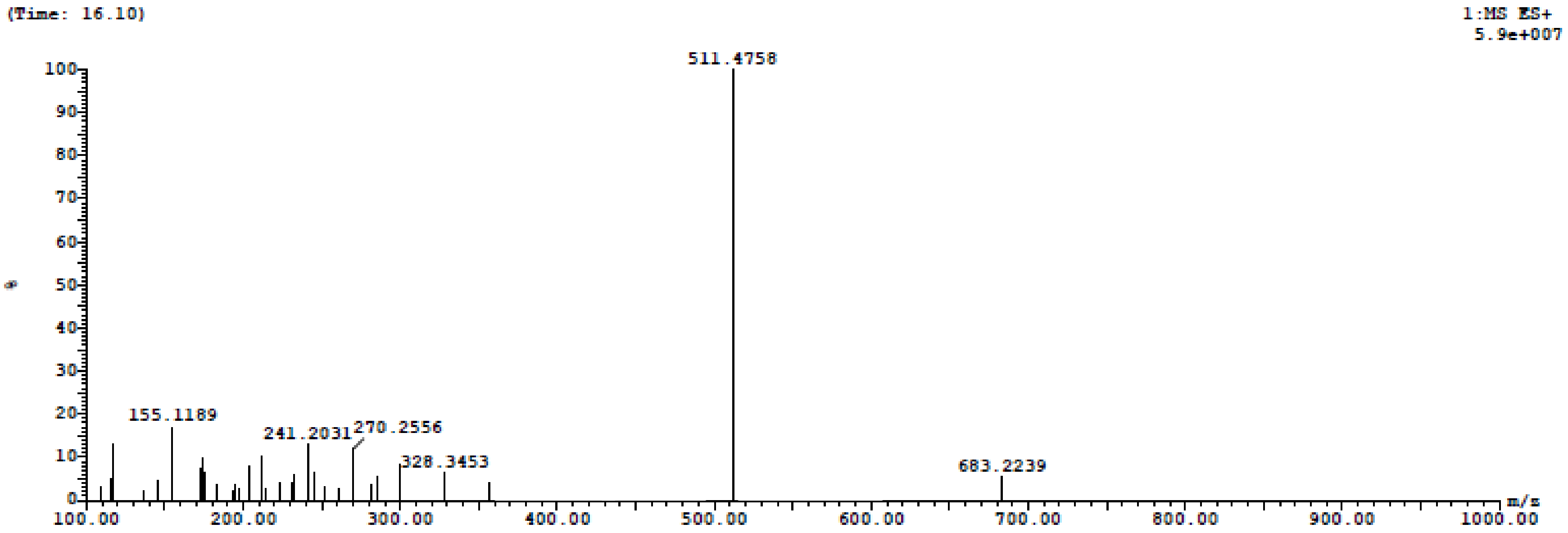

(+) ESI-MS spectrum of Compound **2**
