# Supplementary material for: Nephroprotective effect of Physalis peruviana L. calyx extract and its butanolic fraction against cadmium chloride toxicity in rats and molecular docking of isolated compounds
Source: BMC Complement Med Ther. 2023 Jan 27;23:21. doi: 10.1186/s12906-023-03845-9 (PMC9881262; doi:10.1186/s12906-023-03845-9)
Supplement: Supplementary file 3 — Additional file 3. [file 12906_2023_3845_MOESM3_ESM.pdf]

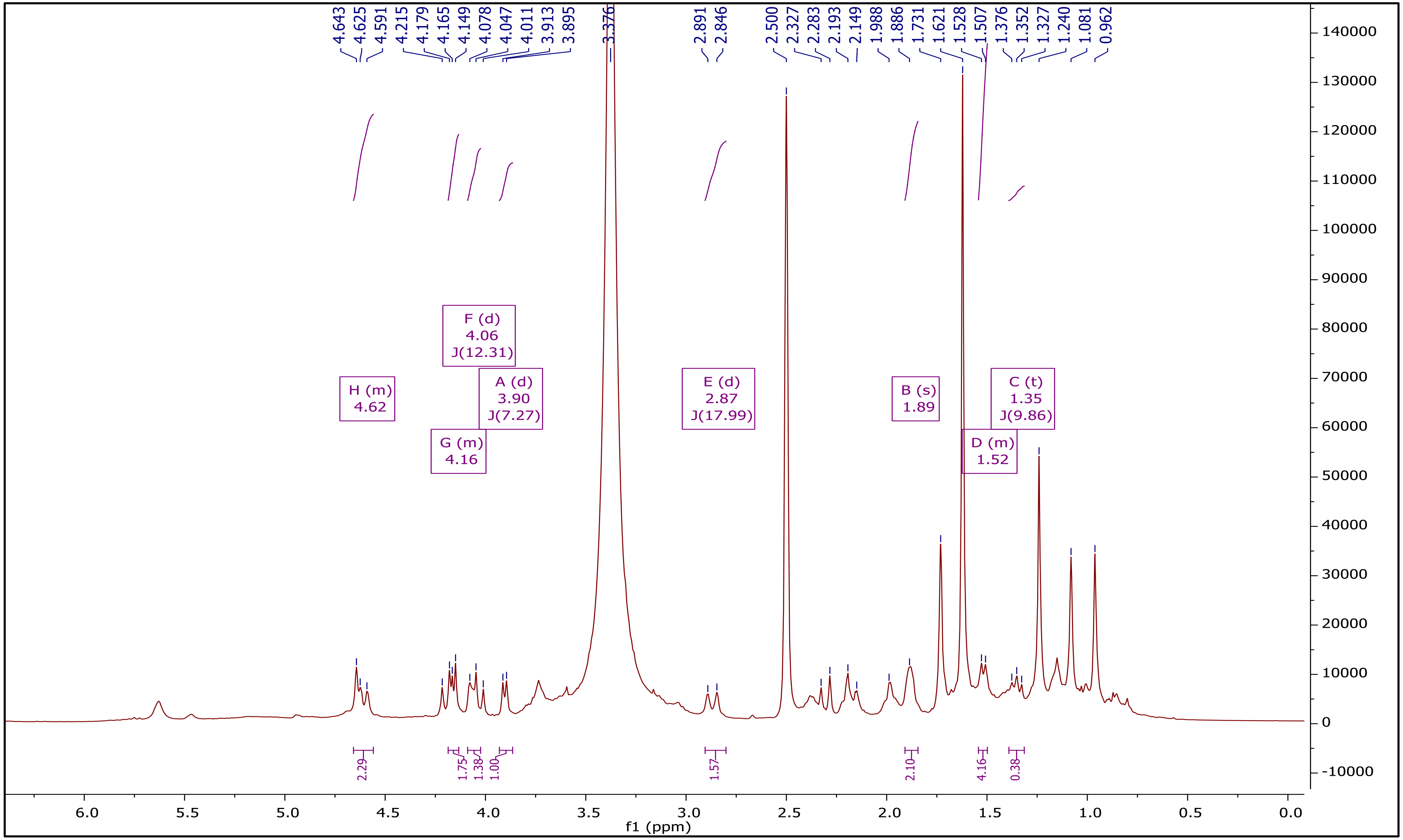


$^{1}$H NMR (400 MHz, DMSO-*d6*) spectrum of compound **3**

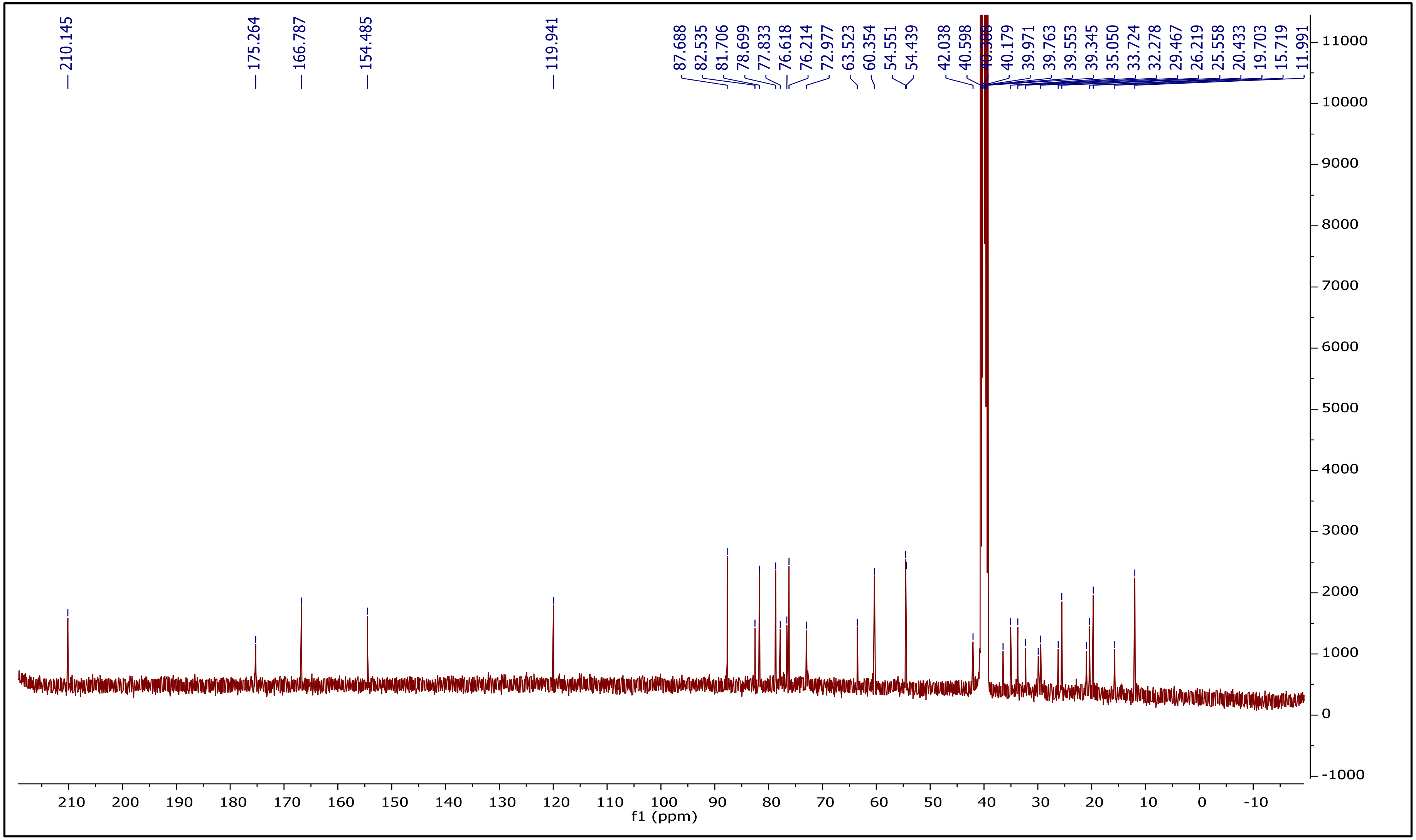


$^{13}$C NMR (100.40 MHz, DMSO-*d6*), spectrum of compound **3**

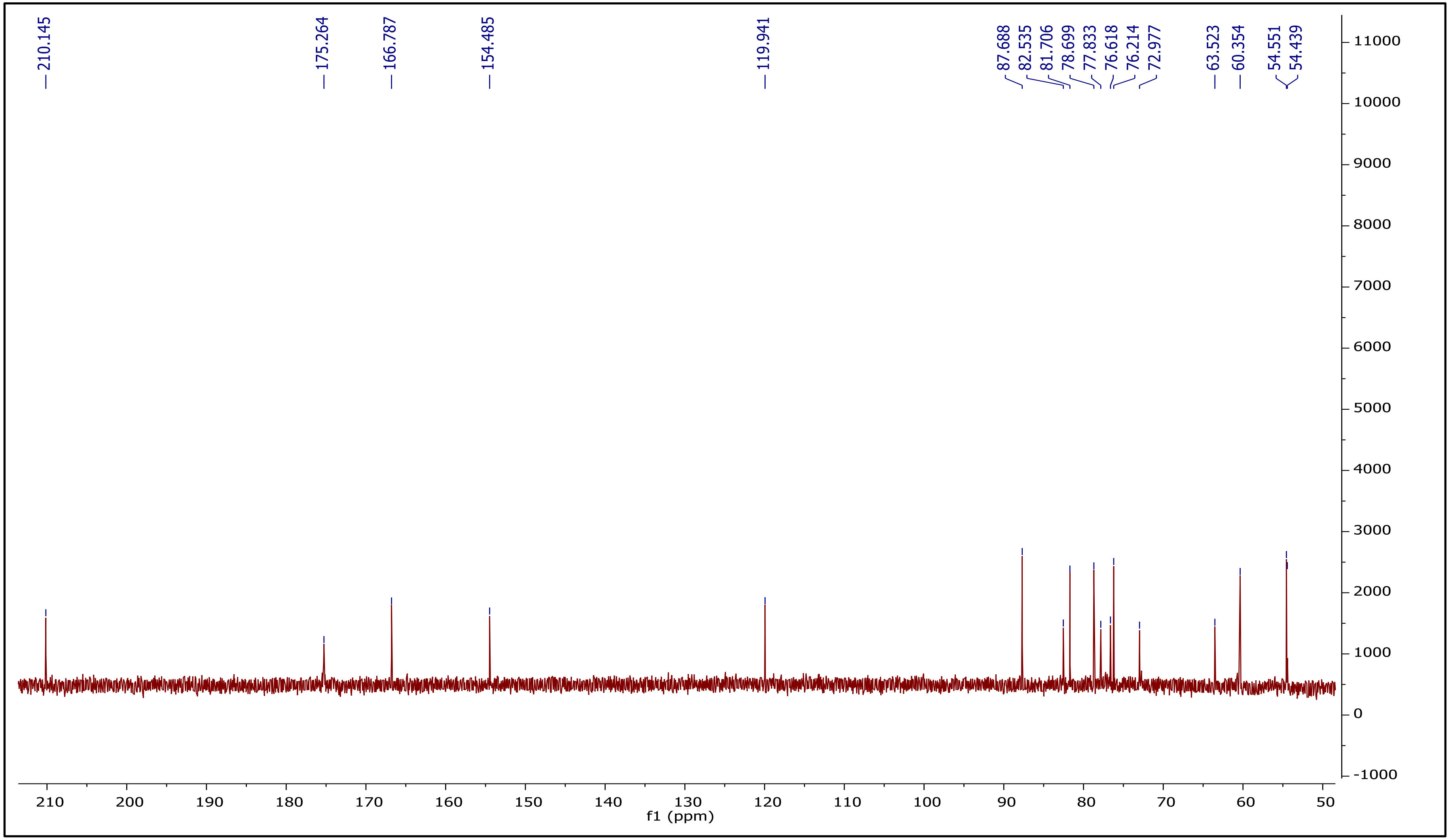


Magnification of $^{13}$C NMR (100.40 MHz, DMSO-*d6*), spectrum of compound **3** (**δ ppm 50 -210**)

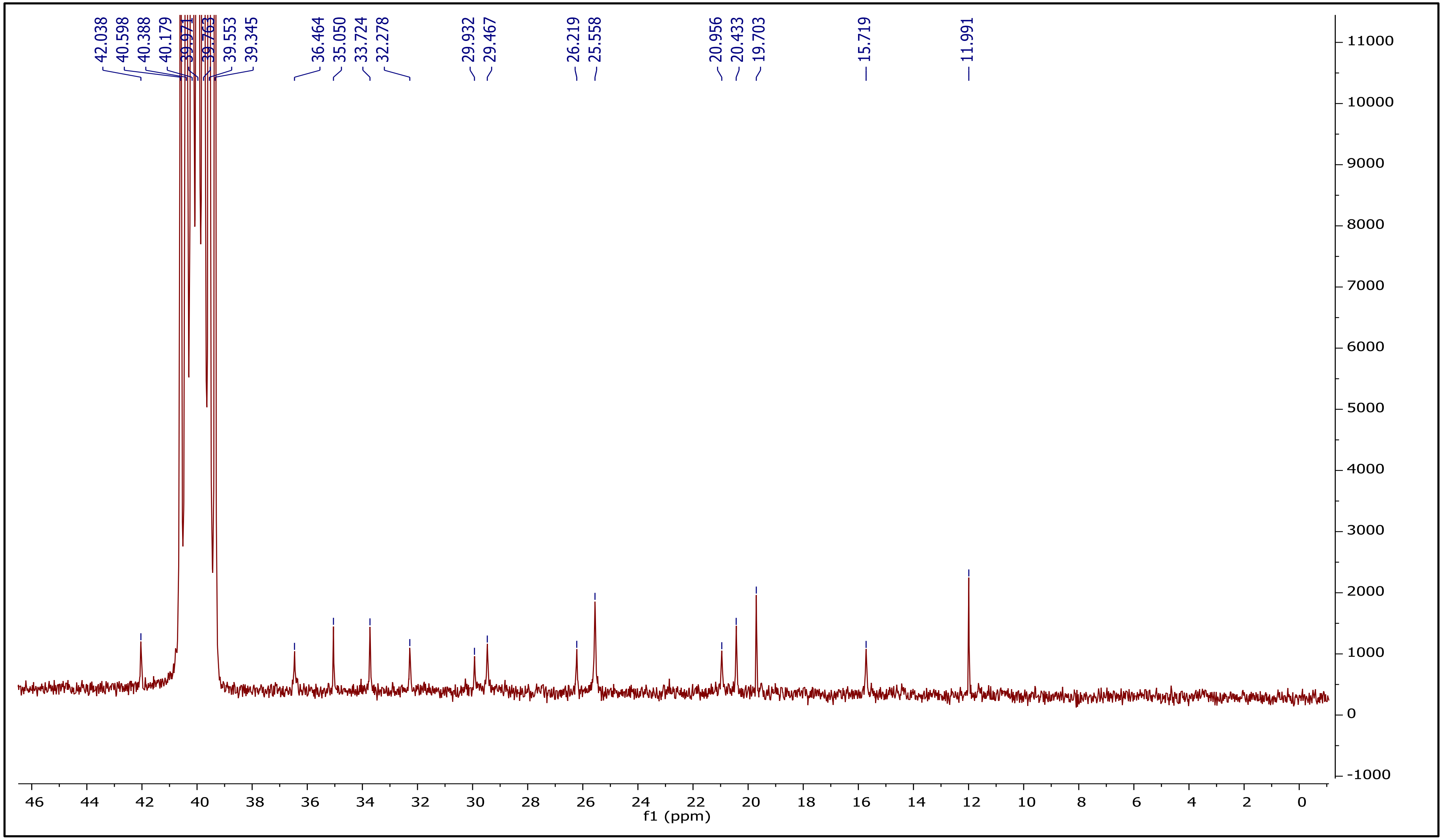


Magnification of $^{13}$C NMR (100.40 MHz, DMSO-*d6*), spectrum of compound **3** (**δ ppm 0 - 46**)

**Center for DRUG DISCOVERY RESEARCH and DEVELOPMENT**
**Openlynx Report -**

Sample: 843 | Vial:1:A,2 | ID:
File:F21 72 | Date:08-Dec-2021 | Time:15:07:08
Description:EB

Printed: Mon Dec 13 13:12:23 2021

| Peak ID | Time | Error PPM |
|---|---|---|
| 82 | 24.03 | |

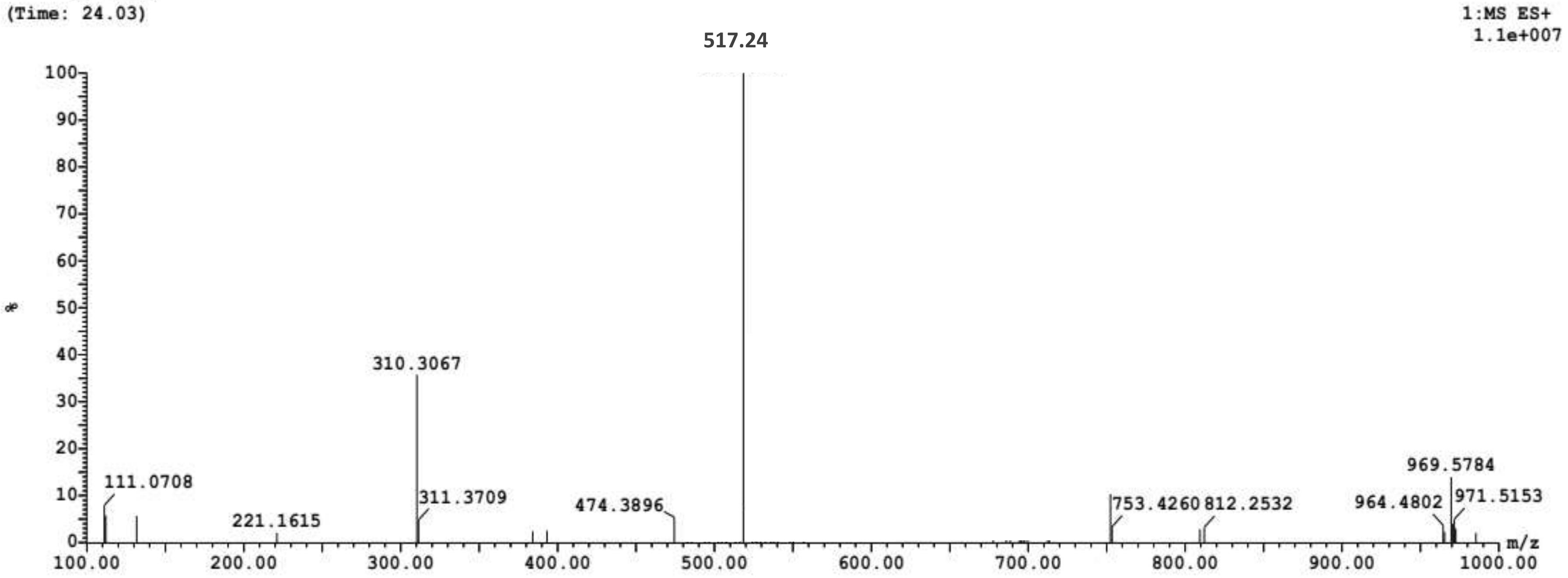


(+) ESI-MS spectrum of Compound **3**
